# Efficacy of an Intelligent and Integrated Older Adult Care Model on Quality of Life Among Home-Dwelling Older Adults: Randomized Controlled Trial

**DOI:** 10.2196/67950

**Published:** 2025-04-21

**Authors:** Rongrong Guo, Jiwen Zhang, Fangyu Yang, Ying Wu

**Affiliations:** 1 School of Nursing, Capital Medical University Beijing China; 2 National Cancer Center/Cancer Hospital, Chinese Academy of Medical Sciences and Peking Union Medical College Beijing China

**Keywords:** efficacy, home care, integrated care, intelligent, elderly people, quality of life, mobile phone

## Abstract

**Background:**

Integrated care models enhanced by the clinical decision support system offer innovative approaches to managing the growing global burden of older adult care. However, their efficacy remains uncertain.

**Objective:**

This study aimed to evaluate the efficacy of an intelligent and integrated older adult care model, termed the SMART (Sensors and scales [receptor], a Mobile phone autonomous response system [central nervous system in the spinal cord], a Remote cloud management center [central nervous system in the brain], and a Total care system [effector]) system, in improving the quality of life (QOL) for home-dwelling older adults.

**Methods:**

In this stratified randomized controlled trial, we consecutively recruited older adults aged 65 years or older from November 1, 2020, to December 31, 2020. Eligible participants were randomly allocated 1:1 to either the SMART group, receiving routine discharge instructions and personalized integrated care interventions across 11 domains (decreased or lost self-care ability, falls, delirium, dysphagia, incontinence, constipation, urinary retention, cognitive decline, depression, impaired skin integrity, and common diseases) generated by the SMART system, or the usual care group, receiving only routine discharge instructions. The intervention lasted for 3 months. The primary end point was the percent change in QOL from baseline to the 3-month follow-up, assessed using the World Health Organization Quality of Life Instrument - Older Adults Module. Secondary end points included functional status at the 3-month follow-up and percent changes in health self-management ability, social support, and confidence in avoiding falling from baseline to the 3-month follow-up. Data were analyzed following the intention-to-treat principle, using covariance or logistic regression models, as appropriate. Subgroup and sensitivity analyses were conducted to assess result consistency and robustness.

**Results:**

In total, 94 participants were recruited, with 48 assigned to the SMART group. The personalized and integrated care by the SMART system significantly improved the QOL among the older adults, with an estimated intervention difference of 11.97% (95% CI 7.2%-16.74%, *P*<.001), and social support and health self-management ability as well, with estimated intervention differences of 6.75% (95% CI 3.19%-10.3%, *P*<.001) and 4.95% (95% CI 0.11%-10%, *P*=.003), respectively, while insignificantly improving in the Modified Falls Efficacy Scale score. Similarly, the SMART system had a 66% reduction in instrumental activities of daily living disability (odds ratio [OR] 0.34, 95% CI 0.11-0.83, *P*=.02). However, the SMART system did not significantly affect activities of daily living disability or the Modified Falls Efficacy Scale score. The subgroup and sensitivity analyses confirmed the robustness of the findings.

**Conclusions:**

The personalized and integrated older adult care by the SMART system demonstrated significant efficacy in improving QOL, health self-management ability, and social support, while reducing instrumental activities of daily living disability among home-dwelling older adults.

**Trial Registration:**

Chinese Clinical Trial Registry ChiCTR-IOR-17010368; https://tinyurl.com/2zax24xr

## Introduction

Recent advances in medicine, public health, and information and communication technology have contributed to increased life expectancy and a rapid global aging trend [1]. In 2021, approximately 963 million individuals aged 65 years and older represented 12.2% of the population worldwide, with projections estimating this number will reach 1.65 billion by 2050, accounting for 16.4% of the total population [2]. Aging often accompanies physiological decline, a higher risk of chronic diseases, and decreased independence in daily activities, leading to rising demand for daily assistance and medical care to maintain an optimal quality of life (QOL) [3]. Despite a strong preference among older adults to be cared for in their homes [4], the current older adult care model fails to address their home-based care needs that span medical and social care domains due to fragmented and inconsistent care services, highlighting the urgent need for a solution that allows older people to age in place while receiving timely and appropriate care for their daily life and diagnosed diseases [5,6].

Integrated care has been proposed as a promising approach to addressing the challenges in older adult care by fostering collaboration among all stakeholders and ensuring continuity in care delivery [7]. The World Health Organization defines integrated care as a person-centered care model that provides comprehensive, coordinated, continuous, and proactive services across various levels and sites of care, encompassing daily life assistance, health promotion, disease prevention, diagnosis, disease management, and rehabilitation throughout the entire lifespan, all tailored to individual needs [8]. By harmonizing the efforts of professional care providers, social workers, and family caregivers, integrated care can minimize redundancies, improve health outcomes, prevent disabilities, and optimize resource allocation, ultimately enhancing the QOL for older adults [9]. Despite the potential benefits, a universally accepted implementation framework for home-based integrated care for older adults remains absent.

Information and communication technology has emerged as a crucial enabler to successfully implement integrated care [10], with the potential to facilitate continuous monitoring, resource integration, seamless information sharing, and timely feedback [11]. For example, Kouroubali and colleagues [12] developed an artificial intelligence (AI)–enabled system to support multidimensional, coordinated, and timely care for older adults with frailty, enabling early detection of frailty, preventing disabilities and adverse events, and reducing hospital admissions [12]. Another example is ProACT, a European digital health platform supporting integrated care for older adults with multimorbidity through symptom monitoring, tailored intervention generation, and data sharing within a care network [13]. However, AI-based solutions often suffer from “black box” issues, where the reasoning processes are difficult to trace or interpret. This lack of transparency can result in inaccurate or conflicting recommendations that may deviate from the established guidelines. In addition, the absence of effective collaborative mechanisms among various care providers complicates the reconciliation of conflicting interests and clarification of responsibilities, limiting care integration.

To address these challenges, a knowledge-based clinical decision support system (CDSS) presents a viable solution [14]. Such a system leverages transparent reasoning processes grounded in pre-embedded knowledge, guidelines, and rules to customize interventions, serving as a useful tool to promote evidence-based, consistent, accurate, and personalized integrated care. In addition, the principle of neural reflex, where receptors collect information and transmit it to the central nervous system for processing, which subsequently sends commands to effectors, can function as an effective collaborative mechanism to promote collaboration among various care providers [15]. This strategy can streamline communication, delineate responsibilities, and enhance coordination, thereby ensuring more coordinated and consistent efforts in older adult care.

Therefore, we developed an intelligent and integrated older adult care model using a knowledge-based CDSS architecture inspired by the principle of neural reflex. Similar to how the neural reflex functions in biological systems, our system was designed to act as the “neural reflex” for older adult care, which consists of Sensors and Scales (receptor), a Mobile Phone Autonomous Response System (central nervous system in the spinal cord), a Remote Cloud Management Center (central nervous system in the brain), and a Total Care System (effector), in short, SMART system.

Although a previous study has confirmed the acceptable usability of the SMART system among older adults, its efficacy in improving outcomes for home-dwelling older people remains unclear. This study aimed to evaluate whether the personalized and integrated care delivered by the SMART system can improve the QOL of older adults living at home through a randomized controlled trial (RCT).

## Methods

### Study Design

This study is a stratified RCT following the tenets of the Declaration of Helsinki. It was prospectively registered in the Chinese Clinical Trial Registry (registration ChiCTR-IOR-17010368) on January 12, 2017. This paper was reported in accordance with the CONSORT (Consolidated Standards of Reporting Trials) guideline (Multimedia Appendix 1) [16].

### Ethical Considerations

The study was conducted in compliance with the ethical principles outlined in the Declaration of Helsinki and approved by the Institutional Review Committee of the Capital Medical University (approval 2015SY49U). Before participating in the study, we provided all potential participants with a comprehensive explanation of the study’s objectives, methods, procedures, and the data to be collected. Written informed consent was obtained from each participant before their enrollment in the study. Participants were assured of their right to withdraw from the study at any time without any penalties or adverse consequences. To safeguard participants’ privacy and confidentiality, all personal identifiers were securely stored in password-protected files. The analysis and reporting of study findings used only deidentified or anonymized data, ensuring that participants’ identities remained confidential. As a token of appreciation for their time and involvement, each participant received a small gift valued at ￥30 (approximately US $4). This reimbursement was intended to acknowledge their contribution while avoiding undue influence on participation decisions.

Every effort was made to ensure that no images or data in the paper or supplementary materials could identify individual participants. In cases where identifiable images were unavoidable, explicit written consent was obtained from the respective participants. No such identifiable images were included in this paper or its supplementary materials.

### Participants

Older adults hospitalized in the Neurology Department at a comprehensive hospital in Beijing, China, were consecutively recruited between November 1, 2020, and December 31, 2020. The Neurology Department, renowned for its advanced diagnostic and therapeutic facilities, serves as a referral center for older adults with diverse neurological conditions. Staffed by highly experienced medical and nursing professionals, the department draws a heterogeneous older adult population from Beijing and surrounding regions. The diversity in socioeconomic backgrounds, education levels, and lifestyle habits provides a rich and representative sample for the study. Individuals were recruited for the study if they (1) were aged 60 years or older; (2) were scheduled for discharge and returning home; (3) had 1 or more diagnosed chronic diseases, or exhibited mild to moderate disability indicated by a Barthel Index between 60 and 100, or had both conditions; (4) were able to communicate; (5) owned an Android (Google)-based smartphone for internet access, as the SMART system is exclusively compatible with Android devices; and (6) expressed a willingness to participate. Older adults were excluded if they (1) were unable to use the SMART system despite repeated instructions; (2) were currently enrolled in any other clinical trials involving investigational products or any other type of medical research judged scientifically or medically incompatible with this study; or (3) had participated in a clinical study and received any treatment, whether active or placebo, within the last 30 days. All participants provided written informed consent on enrollment.

The research team initiated recruitment by thoroughly explaining the study objectives, methods, procedures, potential risks and benefits, and the participants’ rights. Participants were encouraged to ask questions and were given ample time to address any concerns about the study. Once potential participants indicated their willingness to join, they were provided with a written informed consent document. After ensuring that participants fully understood the content and had no remaining questions, they were asked to sign and date the consent form, thereby formally enrolling in the study. To ensure confidentiality, each participant was then assigned a unique identification number, which was used for all subsequent data collection and analysis.

### Smart System

The SMART system is an intelligent and integrated older adult care model. Conceived by a knowledge-based CDSS, it draws inspiration from the principle of neural reflex to facilitate integrated home-based older adult care proactively. An article detailing the system development and usability testing will be published elsewhere.

**Figure 1:**
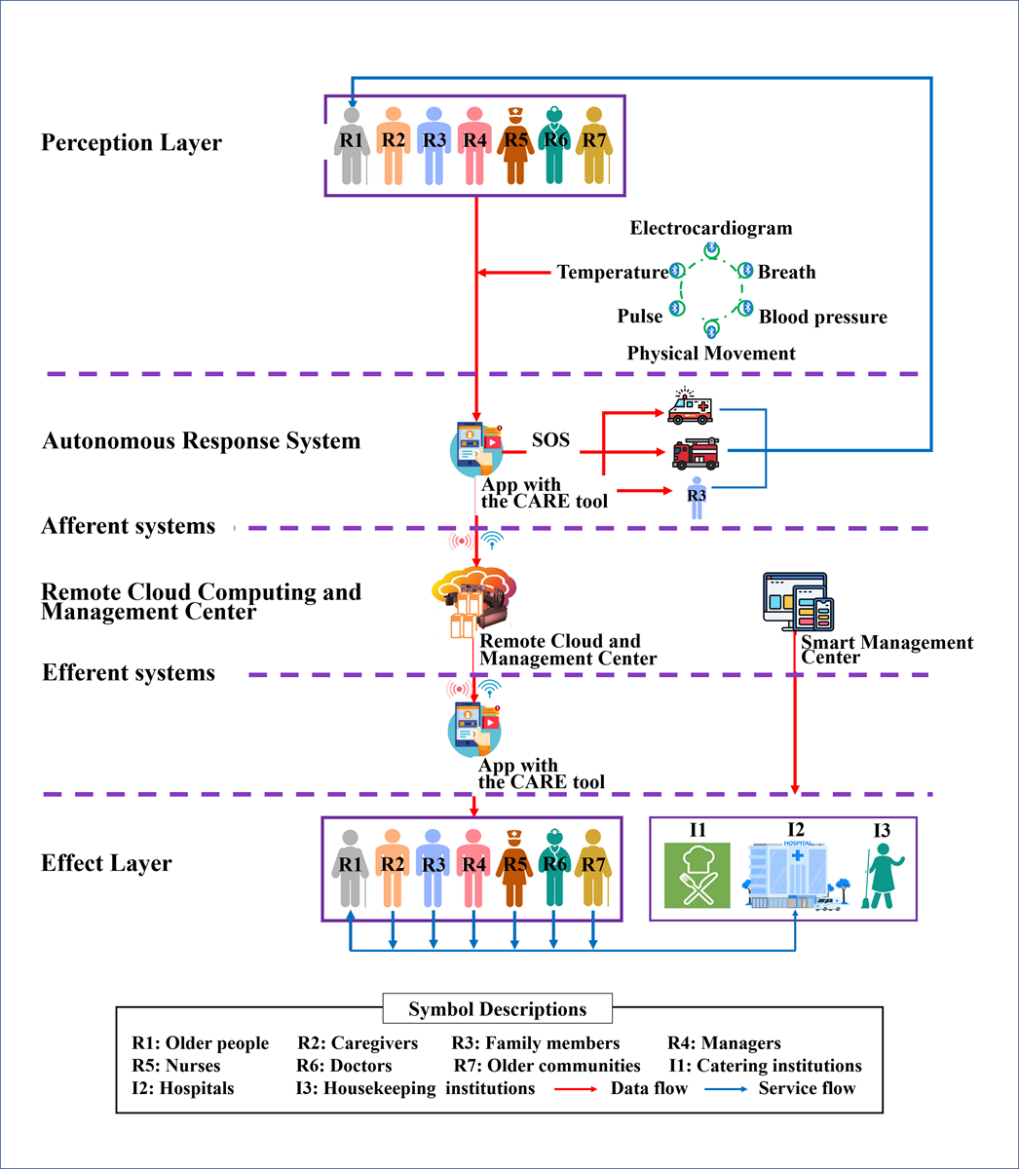
The overall architecture, components, and functional flow of the SMART system. CARE: Continuity Assessment Record and Evaluation. SMART: Sensors and scales (receptor), a Mobile phone autonomous response system (central nervous system in the spinal cord), a Remote cloud management center (central nervous system in the brain), and a Total care system (effector); SOS: A universal distress signal, originally used in Morse code and widely recognized as a call for urgent help in emergencies.

In brief, similar to how the neural reflex functions in biological systems, where receptors collect information and transmit it to the central nervous system for processing before commands are sent to effectors, our SMART system was designed to function as the “neural reflex” for older adult care. It consists of Sensors and Scales (servers as the receptor), a Mobile Phone Autonomous Response System (serves as the central nervous system in the spinal cord), a Remote Cloud Management Center (serves as the central nervous system in the brain), and a Total Care System (serves as the effector, where various care institutions are incorporated to assume responsibility for specific types of care services for older adult, thus promoting the integrated and consistent care). In addition, Wi-Fi and 5G networks serve as afferent nerves or sensory nerves and efferent nerves or motor nerves (Figure 1).

Specifically, the SMART system collects data concerning the overall health status of older adults through real-time monitoring and periodic assessments by the sensors and scales (Multimedia Appendices 2 and 3). The collected data are subsequently uploaded to the Remote Cloud and Management Center via Wi-Fi or 5G networks for comprehensive analysis supported by a foundational knowledge base derived from the latest literature, guidelines, and expert opinions in relevant fields, along with a set of trigger rules. These resources can facilitate accurate diagnoses of the care problems faced by older adults and the generation of customized interventions based on their heterogeneous characteristics in 11 domains, including namely decreased or lost self-care ability, falls, delirium, dysphagia, incontinence, constipation, urinary retention, cognitive decline, depression, impaired skin integrity, and common diseases. After professional review and adjustment by qualified geriatric nurses, these identified care problems and tailored interventions are communicated to caregivers or professional care providers within the total care system as appropriate (Multimedia Appendix 4). The mobile phone autonomous response system uses a set of simple algorithms to handle simple but urgent care problems in the 11 domains, such as first aid for falls.

The SMART system also features reminder functions to encourage older people to adhere to their medication schedules and complete the recommended interventions (Multimedia Appendix 5). Furthermore, the system can deliver health-related information tailored to individual interests by leveraging older people’s login records. In addition, a simple color-block game has been designed to provide entertainment, stimulate mental activity, and enhance cognitive function among older adults.

### Interventions

Participants in the SMART group received customized interventions from the SMART system for 3 months. Trained nurses provided instructions on how to download, install, and navigate the SMART system, ensuring that participants could independently access its various modules. Upon completing the login and registration process, participants were continuously monitored and regularly received customized and integrated care plans presented in texts, diagrams, pictures, and videos. These plans included specific care problems and personalized interventions across 11 domains, such as decreased or lost self-care ability, falls, delirium, dysphagia, incontinence, constipation, urinary retention, cognitive decline, depression, impaired skin integrity, and common diseases. Throughout the intervention, personalized interventions were dynamically adjusted based on user completion status and feedback collected after each delivery. For instance, if an older adult is monitored to have been bedridden for an extended period and not rolled left and right for more than 4 hours, the diagnosed care problem will be “risk for pressure ulcers.” In the case where the older adult has a daily caregiver, the personalized intervention, phrased as “Assist the elderly individual in rolling from side to side and completing the turning record form,” will be delivered to the daily caregiver. Once this intervention is successfully executed, the care problem is considered resolved. Otherwise, the reminder will be continuously pushed until the care intervention is fulfilled. Simultaneously, both the older adult and the daily caregiver will receive health information related to pressure ulcers. Compliance with the intervention was tracked via logs of participants’ login activities, data uploads, and health information downloads recorded by the SMART system. Participants were considered to have good compliance if they used the system at least twice a week, moderate compliance if they accessed it once a week, and poor compliance if they did not engage with the SMART system for more than 2 weeks. Nurses then actively contacted participants with poor compliance to encourage greater engagement with the system. In addition, older adults in the SMART group received routine discharge instructions in accordance with the established standard of care, covering crucial topics such as follow-up appointments, medication adherence, healthy eating, and rehabilitation exercises.

In addition to the similar routine discharge instructions, older adults in the usual care group were granted access to the SMART system, allowing them to only view health-related information without any personalized integrated care interventions over the 3 months.

### End Points

#### Primary End Points

The primary end point was the percent change in QOL from baseline to the 3-month follow-up, which was assessed using the World Health Organization Quality of Life Instrument - Older Adults Module (WHOQOL-OLD). The instrument comprises 24 items from 6 domains, that are sensory abilities (SAB); autonomy (AUT); death and dying (DAD); past, present, and future activities (PPFA); social participation (SP); and intimacy (INT) [17]. Each item is rated on a 5-point Likert scale. The total score obtained by summing up the scores of all items is then converted to a percentage scale as the final score, with higher scores indicating better QOL [18]. The simplified Chinese version of the WHOQOL-OLD demonstrates satisfactory reliability, with Cronbach α coefficients of 0.71-0.84 and intraclass correlation coefficients of 0.77-0.91, acceptable construct validity, and good discriminant validity [19].

#### Secondary End Points

The secondary end points included the functional status at the 3-month follow-up and percent changes in health self-management ability, social support, and confidence in avoiding falling of older adults from baseline to the 3-month follow-up.

Functional status was assessed using the activities of daily living (ADL) scale by Katz et al [20] and the instrumental activities of daily living (IADL) scale by Lawton and Brody [21] at baseline and the 3-month follow-up. The Katz ADL scale consists of 6 dichotomous questions on basic ADLs, including bathing, dressing, feeding, incontinence, toileting, and transfer [20]. The Lawton-Brody IADL scale evaluates participants’ ability to perform instrumental daily activities across 8 areas, which are shopping, food preparation, housekeeping, taking medications, laundering, using telephone, using transportation, and financial management [21]. Responses to both scales are scored dichotomously, with 0 indicating an inability to perform the activity independently or requiring assistance, and 1 indicating independent performance. Summary scores range from 0 (dependent) to 6 (independent) for ADL and from 0 (dependent) to 8 (independent) for IADL. ADL or IADL disability is determined by the presence of at least 1 difficulty in the relevant domains.

Participants’ health self-management ability was assessed using the Rating Scale of Health Self-Management Skill for Adults. This scale comprises 38 items across 3 subscales—behavior, cognition, and environment. Each item is rated on a Likert scale ranging from 1 to 5, and the total score is subsequently converted to a standardized range of 0-100, where a higher score implies greater self-management ability. The scale was validated to have a good reliability and validity in the Chinese context, with a Cronbach α coefficient of 0.93, a split-half reliability index of 0.75, and a content validity index of 0.90, respectively [22].

The Social Support Rating Scale (SSRS) was used to assess the social support of the participants. This instrument, specifically designed for Chinese environment and culture, consists of 10 items spanning 3 dimensions—objective support, subjective support, and utilization of support. Calculated by summing each item score, the total score ranges from 12 to 66. The higher the total score, the better the social support status [23]. The scale has been widely used in Chinese populations with satisfactory reliability and validity [24], indicated by a Cronbach α coefficient of 0.83-0.90 and content validity of 0.72-0.84 [23].

The simplified Chinese version of the Modified Fall Efficacy Scale (MFES) was used to evaluate the confidence in avoiding falls. This self-assessment scale comprises 14 items to quantitatively examine the degree of perceived self-efficacy in avoiding falling during basic activities, ranging from 0 (no confidence) to 10 (absolute confidence) [25]. The average score of each item is regarded as the final MFES score. A lower total score indicates lower confidence and a higher fear of falling. The simplified Chinese version of the MFES has been proven to have good reliability, as evidenced by a Cronbach α coefficient and a split-half reliability of 0.98 and 0.96. It also exhibits satisfactory discriminant and construct validity (all *P*<.001) [26].

#### Safety Assessments

Safety assessments encompassed intervention-emergent adverse events and early discontinuation of the SMART system due to adverse events during the intervention and follow-up periods. Cases of major adverse events and deaths were reviewed by an independent external adjudication committee.

### Randomization and Blinding

Participants were randomly allocated in a 1:1 ratio to either receive customized and integrated care delivered by the SMART system (SMART group) or usual care (usual care group), with stratification based on the Barthel Index (less than 100 or equal to 100). A statistician, who was not involved in data collection or analysis, generated the allocation sequence using computer-generated random numbers. Trained nurses providing the intervention received sequentially numbered, opaque, and sealed envelopes, each containing a card labeled with either the number 1 (indicating integrated care delivered by the SMART system) or 2 (indicating usual care).

Due to the nature of the intervention, neither the older adults nor the nurses providing the intervention could be blinded to group allocation, although they remained unaware of the detailed interventions provided to the other group until study completion. Only the trained investigators were blinded to intervention assignments.

### Sample Size Calculation

The sample size was calculated using PASS software version 2021 (NCSS, LLC). The primary purpose of this study was to demonstrate the superiority of the integrated care delivered by the SMART system over usual care in improving the QOL of older people. Based on previous studies, we anticipated a mean improvement of 8% [27]. To reach 90% power with a significance level (α) set at .05 (2-tailed), a minimum sample size of 31 participants per group was required following the One-Way Analysis of Variance *F* tests, based on a 1:1 allocation ratio. After adjusting for an attrition rate of 20%, the minimum sample size for each group was increased to 39, resulting in a total minimum sample size of 78. The sample size of 78 also provided a power of 90% to demonstrate the superiority concerning other end points, at a 2-sided significance level of .05.

### Statistical Analysis

All data were analyzed based on the intention-to-treat principle. To mitigate the loss of statistical efficiency and bias caused by excluding participants with incomplete data, missing values were imputed 100 times using the method of multiple imputation by chained equations (MICE) based on the same intervention group since Little’s test suggested that data were missing at random (χ^2^_12_=13.8, *P*=.54) [28]. Any missing categorical variables were dichotomized following the MICE imputation.

Continuous variables were presented as mean with SD or median with IQRs (25% percentile, 75% percentile), as appropriate. Between-group comparisons for continuous variables were performed using either the Student *t* test or the Mann-Whitney *U* test. Categorical variables were expressed as frequencies or proportions (%), with comparisons conducted using chi-square, Fisher exact test, or Wilcoxon rank-sum test. For the continuous end points, a covariance model including randomized group and stratification factor as fixed effects and baseline measure as a covariate was used [29]. Categorical end points were analyzed using a logistic regression model with the same fixed effects and covariates as continuous end points, where treatment difference was assessed by odds ratios (ORs). The types and measurement methods of all covariates and end points, along with the regression models, were presented in Table S1 in Multimedia Appendix 6.

To evaluate whether baseline characteristics could influence the superiority of the integrated care delivered by the SMART system over usual care in improving the QOL for older adults, subgroup analyses were performed by age group (60-69 y and >70 y), sex (male and female), and BMI group (normal [18.5-23.9 kg/m^2^], abnormal [<18.5 kg/m^2^ or ≥24.0 kg/m^2^]) [30]. In addition, we conducted several sensitivity analyses to assess the stability of our findings. First, we repeated the analyses on continuous end points by using the change values as a measurement approach. Second, we performed a per-protocol analysis by only including participants who adhered to the study protocol to avoid inaccurate estimation of the improvement of end points.

The statistical analyses were performed using RStudio version 4.2.0 (Posit BBC). Statistical significance was set at a 2-sided *P*<.05.

## Results

### Study Participants and Baseline Characteristics

**Figure 2:**
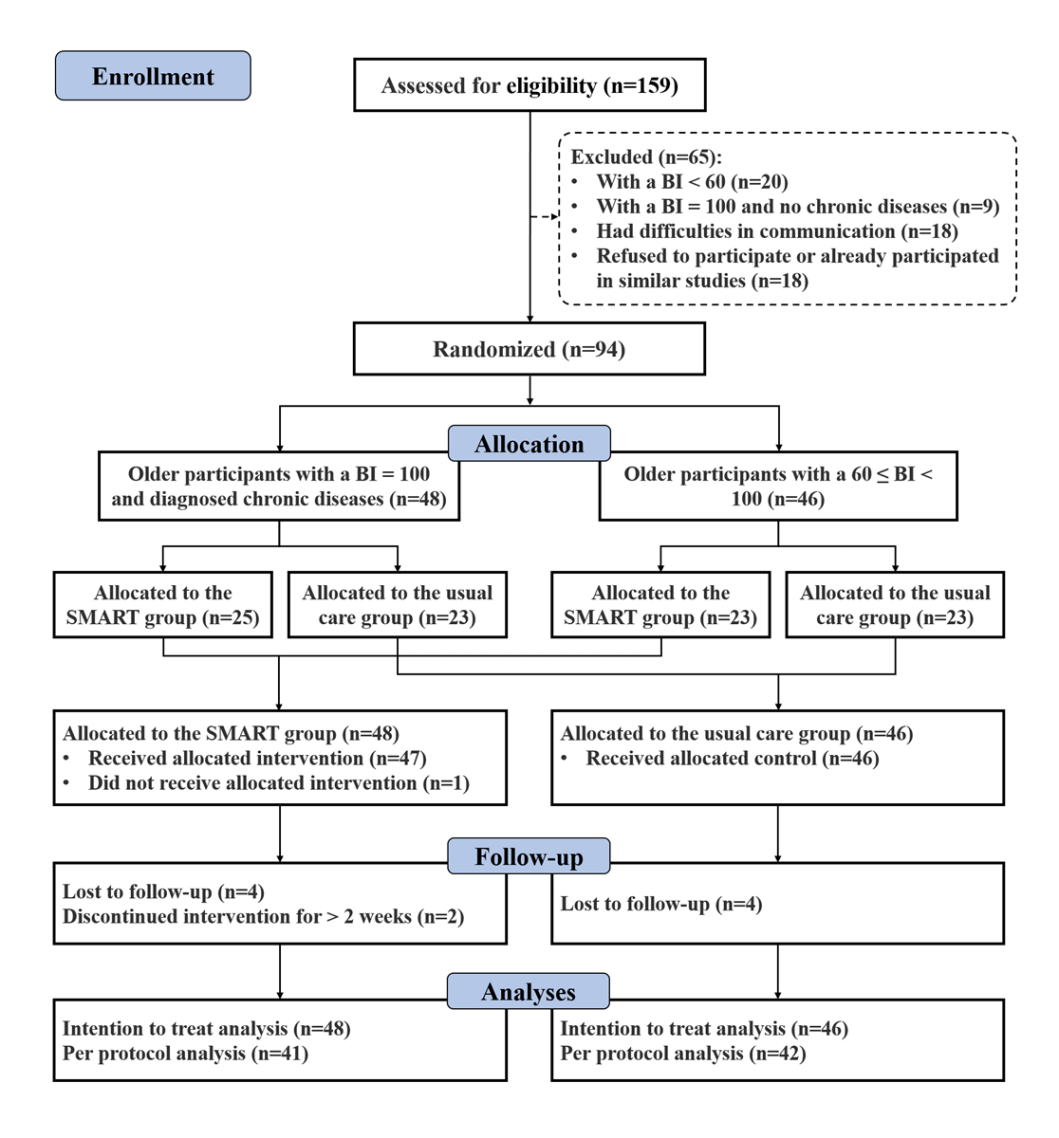
The flow diagram of older adult individuals. BI: Barthel Index. SMART: Sensors and scales (receptor), a Mobile phone autonomous response system (central nervous system in the spinal cord), a Remote cloud management center (central nervous system in the brain), and a Total care system (effector).

The inclusion process of the study participants is presented in Figure 2. Between November 1, 2020, and December 31, 2020, a total of 159 older adults were screened for eligibility and 94 were randomly allocated to the usual care group (n=46) or SMART group (n=48). Out of the 94 older adults, a total of 83 (88%) participants rigorously completed the predefined intervention.

Baseline characteristics of older adults are summarized in Table 1. The mean age of older adults in the SMART group was 69.50 (SD 6.53) years old with 60% (29/48) being male, while older adults in the usual care group were on average 70.83 (SD 7.11) years old with 61% (28/46) being male. In both the SMART and usual care groups, the majority of older adults were married and lived with others. Overall, baseline characteristics were similar across the 2 groups.

**Table 1:** Baseline characteristics of older adults in the intention-to-treat analysis (randomized controlled trial conducted from November 1 to December 31, 2020).

Variables		SMART^a^ Group (n=48), n (%)	Usual care group (n=46), n (%)	*t* test (*df*), *z* score, or chi-square (*df*)	*P* value
Age (y), mean (SD)		69.50 (6.53)	70.83 (7.11)	0.94 (92)^b^	.35
Male		29 (60)	28 (61)	0.01 (1)^c^	.96
BMI (kg/m^2^), mean (SD)		24.34 (3.87)	23.29 (2.48)	–1.56 (92)^b^	.12
**Marital status**				—^d,e^	.20
	Married	32 (67)	37 (80)		
	Widowed	15 (31)	9 (20)		
	Divorced	1 (2)	0 (0)		
**Education**				1.57^f^	.67
	Primary school and below	9 (19)	5 (11)		
	Junior high school	13 (27)	15 (33)		
	Senior high or vocational school	17 (35)	15 (33)		
	College and above	9 (19)	11 (24)		
**Dwelling status**				—^d,e^	.50
	Living with others	46 (96)	45 (98)		
	Living alone	2 (4)	0 (0)		
	Nursing home	0 (0)	1 (2)		
Visual impairment		19 (40)	17 (37)	0.07 (1)^c^	.79
Hearing impairment		17 (35)	16 (35)	0.004 (1)^c^	.95
Smoking		14 (29)	9 (20)	1.17 (1)^c^	.28
Alcohol drinking		10 (21)	6 (13)	1.01 (1)^c^	.32

^a^SMART: Sensors and scales (receptor), a Mobile phone autonomous response system (central nervous system in the spinal cord), a Remote cloud management center (central nervous system in the brain), and a Total care system (effector).

^b^Student *t* test.

^c^Chi-square test.

^d^Fisher exact test.

^e^Not applicable.

^f^Wilcoxon rank-sum test.

### Primary End Points

**Figure 3:**
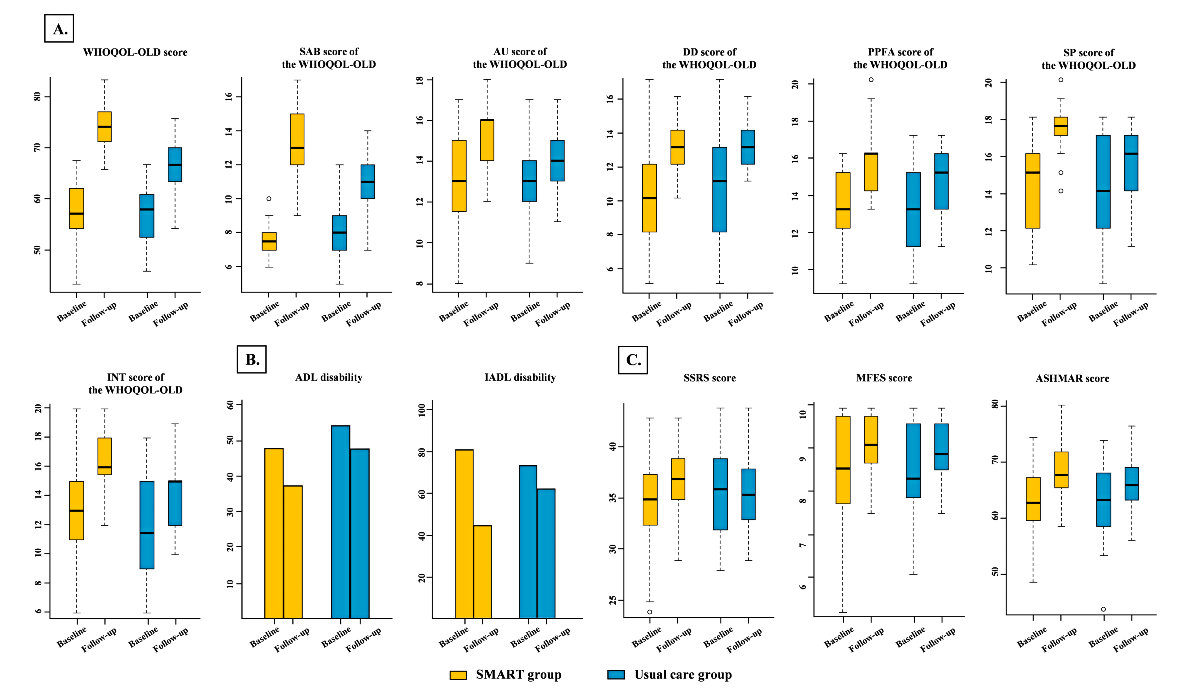
Effects of the personalized and integrated care provided by the SMART system combined with usual care versus usual care on the primary and secondary endpoints in the intention-to-treat analysis. (A) Effects of the personalized and integrated care provided by the SMART system combined with usual care versus usual care on quality of life in the intention-to-treat analysis. (B) Effects of the personalized and integrated care provided by the SMART system combined with usual care versus usual care on functional status in the intention-to-treat analysis. (C) Effects of the personalized and integrated care provided by the SMART system combined with usual care versus usual care on other secondary outcomes in the intention-to-treat analysis. ADL: Activities of Daily Living; AHSMSRS: The Rating Scale of Health Self-Management Skill for Adults; DAD: Death and Dying; IADL: Instrumental Activities of Daily Living; INT: Intimacy; MFES: Modified Fall Efficacy Scale; PPFA: Past, Present, and Future Activities; SAB: Sensory Abilities; AUT, Autonomy; SMART: Sensors and scales (receptor), a Mobile phone autonomous response system (central nervous system in the spinal cord), a Remote cloud management center (central nervous system in the brain), and a Total care system (effector); SP: Social Participation; SSRS: Social Support Rating Scale; WHOQOL-OLD: World Health Organization Quality of Life Instrument-Older Adults Module.

The analysis results of the primary end points are presented in Table 2 and Figure 3A. The mean percent change in WHOQOL-OLD score from baseline to the 3-month follow-up was 29.56% (95% CI 25.83%-33.26%) in the SMART group and 17.59% (95% CI 14.48%-20.69%) in the usual care group, respectively. The covariance model demonstrates a statistically significant superiority of the integrated care delivered by the SMART system in improving the QOL, with an estimated intervention difference of 11.97% (95% CI 7.2%-16.74%, *P*<.001). Specifically, within the 6 dimensions of the WHOQOL-OLD scale, the interventions delivered through the SMART system resulted in significant improvements in the SAB, AUT, PPFA, and SP scores for older adults (all *P*<.001). However, no significant enhancements were observed in the INT and DAD scores (*P*=.63 and *P*=.58, respectively).

**Table 2:** Primary and secondary end points, along with the estimated differences observed in the intention-to-treat analysis.

End points				SMART^a^ Group (n=48)		Usual care group (n=46)		Difference, mean (95% CI)^b^		OR^c^ (95% CI)		*P* value
**Primary end points, mean (95% CI)**												
	Percent change^d^ in WHOQOL-OLD^e^ score (%)			29.56 (25.85 to 33.26)		17.59 (14.48 to 20.69)		11.97 (7.20 to 16.74)		—^f^		<.001^g^
	Percent change^d^ in SAB^h^ score of the WHOQOL-OLD scale (%)			78.11 (67.27 to 88.96)		40.45 (31.57 to 49.34)		37.66 (23.82 to 51.49)		—		<.001^g^
	Percent change^d^ in AUT^i^ score of the WHOQOL-OLD scale (%)			20.48 (14.29 to 26.68)		10.71 (6.09 to 15.33)		9.77 (2.15 to 17.40)		—		<.001^g^
	Percent change^d^ in DAD^j^ score of the WHOQOL-OLD scale (%)			40.51 (26.44 to 54.58)		37.94 (20.31 to 55.57)		2.57 (–19.69 to 24.82)		—		.58
	Percent change^d^ in PPFA^k^ score of the WHOQOL-OLD scale (%)			20.05 (14.44 to 25.66)		10.73 (6.58 to 14.87)		9.33 (2.44 to 16.21)		—		<.001^g^
	Percent change^d^ in SP^l^ score of the WHOQOL-OLD scale (%)			26.93 (19.74 to 34.11)		13.43 (8.99 to 17.87)		13.50 (5.16 to 21.83)		—		<.001^g^
	Percent change^d^ in INT^m^ score of the WHOQOL-OLD scale (%)			34.73 (24.08 to 45.38)		32.61 (19.95 to 45.26)		2.12 (–14.20 to 18.44)		—		.63
**Secondary end points**												
	**Functional status**											
		Participants with ADL^n^ disability at 3-month follow-up, n (%)	18 (38)		22 (48)		–10.33 (–30.49 to 9.84)		0.54 (0.12 to 2.16)		.39	
		Participants with IADL^o^ disability at 3-month follow-up, n (%)	21 (44)		28 (61)		–17.12 (–37.28 to –3.04)		0.34 (0.11 to 0.83)		.02^g^	
	Percent change^d^ in SSRS^p^ score (%), mean (95% CI)			6.94 (3.93 to 9.95)		0.19 (–1.79 to 2.18)		6.75 (3.19 to 10.30)		—		<.001^g^
	Percent change^d^ in MFES^q^ score (%), mean (95% CI)			8.39 (4.73 to 12.06)		5.51 (3.07 to 7.95)		2.88 (–1.47 to 7.22)		—		.17
	Percent change^d^ in ASHMAR^r^ score (%), mean (95% CI)			10.60 (7.14 to 14.07)		5.66 (1.88 to 9.44)		4.95 (0.11 to 10.00)		—		.003^g^

^a^SMART: Sensors and scales (receptor), a Mobile phone autonomous response system (central nervous system in the spinal cord), a Remote cloud management center (central nervous system in the brain), and a Total care system (effector).

^b^Data are absolute differences between mean changes and expressed in percentage points.

^c^OR: odds ratio.

^d^The percent change values are presented as mean (95% CI) values.

^e^WHOQOL-OLD: World Health Organization Quality of Life Instrument-Older Adults Module.

^f^Not applicable.

^g^Indicate statistically significant variables (*P*<.05).

^h^SAB: sensory abilities.

^i^AUT: autonomy.

^j^DAD: death and dying.

^k^PPFA: past, present, and future activities.

^l^SP: social participation.

^m^INT: intimacy.

^n^ADL: activities of daily living.

^o^IADL: instrumental activities of daily living

^p^SSRS: Social Support Rating Scale.

^q^MFES: Modified Fall Efficacy Scale.

^r^AHSMSRS: The Rating Scale of Health Self-Management Skill for Adults.

### Secondary End Points

Regarding the functional status of older adults (Figure 3B and Table 2), the integrated care delivered by the SMART system significantly reduced IADL disability compared with usual care (OR 0.34, 95% CI 0.11-0.83, *P*=.02), while no statistically significant reduction in ADL disability was observed (*P*=.39).

As presented in Figure 3C and Table 2, the improvement in the SSRS score was significantly greater in the SMART group compared with the usual care group, with an estimated intervention difference of 6.75% (95% CI 3.19%-10.3%, *P*<.001). Specifically, the SMART group exhibited a change of 6.94% (95% CI 3.93%-9.95%), while the usual care group reflected only a minimal change of 0.19% (95% CI 1.79%-2.18%) from baseline to the 3-month follow-up. Similarly, the integrated care delivered by the SMART system resulted in a substantial improvement of 4.95% (95% CI 0.11%-10%, *P*=.003) in the ASHMAR score among older adults compared with the usual care group. The improvement levels were 10.6% (95% CI 7.14%-14.07%) for the SMART group versus 5.66% (95% CI 1.88%-9.44%) for the usual care group. Despite these favorable secondary end points, the SMART group demonstrated a statistically nonsignificant improvement in the MFES score from baseline to the 3-month follow-up (an estimated intervention difference of 2.88%, 95% CI –1.47% to 7.22%, *P*=.17), with the respective improvement levels of the SMART group and the usual care group being 8.39% (95% CI 4.73%-12.06%) and 5.51% (95% CI 3.07%-7.95%).

### Intervention Compliance and Adverse Events

Among the older adults in the SMART group, 12 (25%) demonstrated good compliance with the SMART interventions, 27 (56%) had moderate compliance, and 9 (19%) had poor adherence.

During the 3-month intervention period, no adverse events were reported in either the SMART group or the usual care group.

### Subgroup and Sensitivity Analyses

As summarized in Tables S2-S12 and Figures S1-S7 in the Multimedia Appendix 6, consistent results were observed in both primary and secondary end points across the subgroup and sensitivity analysis, despite the integrated care delivered by the SMART system not demonstrating a significant reduction in IADL disability among older adults aged 60-69 years and females, which verified the robustness of our findings to a certain extent.

## Discussion

### Principal Results

This study demonstrates a significant improvement in the QOL of home-dwelling older adults who received personalized and integrated care provided by the SMART system, an intelligent and integrated older adult care model that facilitates integrated home-based older adult care, compared with those receiving usual care. In addition, substantial enhancements were also observed in health self-management ability and social support, along with a significant decrease in IADL disability. To the best of our knowledge, this represents the first RCT to evaluate the efficacy of personalized and integrated care service delivered through a digitally structured system for home-dwelling older adults.

Our findings exhibit both face and internal validity for the following reasons. First, in line with previous studies [31,32], this study confirms the positive effect of personalized, intelligent, and integrated care interventions on improving outcomes of older adults, particularly the QOL. Furthermore, our research design employs an RCT, recognized as the gold standard for establishing causality [33]. The random assignment of participants minimizes selection bias and controls for confounding variables, ensuring that the observed outcome differences can be attributed directly to the SMART system interventions. Furthermore, the use of standardized interventions and measures throughout the study enhances the reliability and validity of our findings. Finally, the sensitivity analyses yield consistent results, further supporting the reliability and generalizability of the research findings.

Through a 3-month intervention, our study demonstrated that personalized and integrated care significantly enhanced the QOL, health self-management ability, and social support, while also reducing IADL disability among older adults living at home. Several reasons may account for these outcomes. First, as a knowledge-based CDSS, the SMART system can deliver integrated care interventions tailored to the specific needs and preferences of older adults in easily understandable formats, such as text, images, and videos. This customization and ease of use fosters trust and engagement with the recommended interventions, promoting a sense of ownership and empowerment that leads to improved health outcomes in the QOL and IADL functioning [34,35]. Second, the SMART system creates a supportive feedback environment that transcends time and space limitations [36]. It continuously adjusts interventions based on real-time monitoring and provides timely encouragement and reminders, which allows older adults to focus on the recommended interventions, thus greatly promoting proactive health behaviors and helping manage their conditions more effectively. Third, the personalized interventions and health-related information provided by the SMART system likely contribute to the significant improvements observed in participants’ health self-management ability. Fourth, the SMART system can facilitate social connections through online platforms, reducing feelings of isolation and enhancing social support networks, which in turn contributes to the improved QOL [37].

However, the SMART system did not yield significant improvements in fall efficacy or effectively reduce ADL disability among older adults. One potential explanation is that the short intervention duration was insufficient to produce noticeable effects in these critical outcomes. Furthermore, the complexity of multiple intervention components could have hindered some participants from fully comprehending or implementing the care interventions, thereby reducing the overall efficacy [38]. Another important factor to consider is the relatively low participant compliance. Inadequate engagement may have curtailed adherence to the care interventions, limiting the potential benefits. Future studies with a longer intervention duration, simplified intervention components, and tailored strategies to enhance participant engagement may be necessary to foster significant improvements in ADL disability and fall efficacy among this population.

Regarding the subgroup analyses, no significant reduction in IADL disability was observed among older women and individuals aged 60-69 years after receiving the personalized and integrated care delivered by the SMART system. One possible explanation is that women were found to exhibit lower digital literacy, making it more difficult for them to adapt to the new strategies introduced by the SMART system, thereby affecting their responses to these interventions [39]. In addition, given that individuals aged 60-69 years typically maintain relatively good functional capacity, the potential for improvement may be limited, which could restrict the efficacy of any intervention [40]. Finally, the short intervention period may not allow sufficient time for participants to fully integrate the interventions into their daily routines or to experience noticeable improvements.

This study is the first RCT exploring the efficacy of personalized and integrated care on older adults living at home. The positive findings provide valuable evidence to support the potential use of similar systems in clinical practice and offer insights for the future development of such systems. By demonstrating the efficacy of integrated care facilitated by our SMART system, the study also emphasizes the importance of tailoring interventions to meet older people’s specific needs, which can in turn lead to better health outcomes and enhanced functional status, ultimately improving the QOL.

### Limitations

There are several limitations in this study. First, our study recruited a relatively small sample size; however, it provided sufficient statistical power to demonstrate the efficacy of the SMART system. Second, our study was inherently limited to finite representativeness by a short intervention period, leaving some long-term changes undetected. This limitation could explain why certain secondary end points, such as the ADL disability reduction and percent change in the MFES score, did not show statistically significant changes. A larger sample size with a long intervention period is therefore required to further validate our findings for wider generalization. Third, due to the challenges presented by the COVID-19 pandemic in accessing older adults’ homes, we recruited older adults who were about to return home after discharge. This approach may introduce a potential selection bias, as hospitalized older adults are more likely to possess lower levels of digital divide, receive assistance from health care professionals, and exhibit higher compliance with the personalized interventions, all of which could influence our findings and the study generalizability. Fourth, the lack of blinding older adults and the nurses administering the intervention may introduce potential performance bias, although they remained unaware of the specific interventions provided to the other group until the study was completed. To reduce the risk of bias, other procedures such as proper allocation concealment were implemented to ensure rigor and reproducibility [41]. Fifth, although we primarily relied on self-reported ratings for efficacy evaluation, using percent changes as a metric for measuring outcomes provided a more objective assessment and mitigated potential biases effectively [42]. Finally, although this study has explored the efficacy of the SMART system under controlled conditions, it did not address the challenges that may arise when implementing the SMART system in real-world settings. These challenges primarily manifest in several areas such as the financial burden associated with maintaining and updating the foundational knowledge base and the iterative upgrades of the SMART system, the additional workload placed on geriatric nurses, the digital literacy and system acceptance of older adults and their family members, the risks related to data security and privacy violations, ethical and moral considerations, insufficient policy adaptability, and difficulties in coordinating with various care service providers. The absence of exploration of these aspects results in an unclear understanding of the feasibility and sustainability of the SMART system in practical applications. Therefore, more comprehensive real-world research is essential to bridge the gap between controlled conditions and actual practice and to develop appropriate solutions.

### Conclusions

This study demonstrated a significant improvement in the QOL of older adults living at home after receiving personalized and integrated care provided by the SMART system. Future RCTs with large sample sizes and long intervention periods are needed to validate their efficacy in the Chinese older adults.

## Appendix

Multimedia Appendix 1 CONSORT-eHEALTH checklist (V 1.6.1).

Multimedia Appendix 2 Geriatric assessment module.

Multimedia Appendix 3 Daily monitoring module.

Multimedia Appendix 4 Medication management module.

Multimedia Appendix 5 Professional review and information reminder module.

Multimedia Appendix 6 Detailed imputation methods, subgroup analysis results, and sensitivity analysis results.

## Abbreviations

ADL: activities of daily living
AI: artificial intelligence
AUT: autonomy
CDSS: clinical decision support system
CONSORT: Consolidated Standards of Reporting Trials
DAD: death and dying
IADL: instrumental activities of daily living
INT: intimacy
MFES: Modified Fall Efficacy Scale
MICE: multiple imputation by chained equations
OR: odds ratio
PPFA: past, present, and future activities
QOL: quality of life
RCT: randomized controlled trial
SAB: sensory abilities
SMART: Sensors and scales (receptor), a Mobile phone autonomous response system (central nervous system in the spinal cord), a Remote cloud management center (central nervous system in the brain), and a Total care system (effector)
SP: social participation
SSRS: Social Support Rating Scale
WHOQOL-OLD: World Health Organization Quality of Life Instrument - Older Adults Module

## Data Availability

The datasets generated or analyzed during this study are not publicly available due to ethical and legal restrictions but are available from the corresponding author on reasonable request.
