# Supplementary material for: Efficacy of an Intelligent and Integrated Older Adult Care Model on Quality of Life Among Home-Dwelling Older Adults: Randomized Controlled Trial
Source: J Med Internet Res. 2025 Apr 21;27:e67950. doi: 10.2196/67950 (PMC12053148; doi:10.2196/67950)
Supplement: Multimedia Appendix 1 [file jmir_v27i1e67950_app1.docx]

**
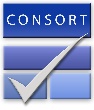
CONSORT-EHEALTH checklist (V1.6.1): Information to include when reporting ehealth/mhealth trials**

| **Section/Topic** | **Item No.** | **CONSORT* Checklist Item** | **Reported on page No** |
| --- | --- | --- | --- |
| **Title and abstract** | | | |
|  | 1a | Identification as a randomized trial in the title | Page 1 |
|  | 1b | Structured summary of trial design, methods, results, and conclusions (NPT** extension: Description of experimental treatment, comparator, care providers, centers, and blinding status) | Pages 1 & 2 |
| **Introduction** | | | |
| Background and objectives | 2a | Scientific background and explanation of rationale | Pages 2 & 3 |
|  | 2b | Specific objectives or hypotheses | Page 3 |
| **Methods** | | | |
| Trial design | 3a | Description of trial design (such as parallel, factorial) including allocation ratio | Page 3 |
|  | 3b | Important changes to methods after trial commencement (such as eligibility criteria), with reasons | Not applicable |
| Participants | 4a | Eligibility criteria for participants | Pages 4 & 5 |
|  | 4b | Settings and locations where the data were collected | Pages 4 & 5 |
| Interventions | 5 | The interventions for each group with sufficient details to allow replication, including how and when they were actually administered | Pages 4-8 |
| Outcomes | 6a | Completely defined pre-specified primary and secondary outcome measures, including how and when they were assessed | Pages 9 & 10 |
|  | 6b | Any changes to trial outcomes after the trial commenced, with reasons | Not applicable |
| Sample size | 7a | How sample size was determined (NPT: When applicable, details of whether and how the clustering by care providers or centers was addressed ) | Pages 10 & 11 |
|  | 7b | When applicable, explanation of any interim analyses and stopping guidelines | Not applicable |
| Randomization |  |  |  |
| Sequence generation | 8a | Method used to generate the random allocation sequence (NPT: When applicable, how care providers were allocated to each trial group) | Page 10 |
|  | 8b | Type of randomisation; details of any restriction (such as blocking and block size) | Page 10 |
| Allocation concealment mechanism | 9 | Mechanism used to implement the random allocation sequence (such as sequentially numbered containers), describing any steps taken to conceal the sequence until interventions were assigned | Page 10 |
| Implementation | 10 | Who generated the random allocation sequence, who enrolled participants, and who assigned participants to interventions | Page 10 |
| Blinding | 11a | If done, who was blinded after assignment to interventions (for example, participants, care providers, those assessing outcomes) and how (NPT: Whether or not administering co-interventions were blinded to group assignment) | Page 10 |
|  | 11b | If relevant, description of the similarity of interventions | Not applicable |
| Statistical methods | 12a | Statistical methods used to compare groups for primary and secondary outcomes (NPT: When applicable, details of whether and how the clustering by care providers or centers was addressed) | Pages 11 |
|  | 12b | Methods for additional analyses, such as subgroup analyses and adjusted analyses | Page 11 |
| Ethics & Informed Consent | X26 | *(not a CONSORT item)* | Pages 3 & 4 |
| **Results** | | | |
| Participant flow (a diagram is strongly recommended) | 13a | For each group, the numbers of participants who were randomly assigned, received intended treatment, and were analysed for the primary outcome (NPT: The number of care providers or centers performing the intervention in each group and the number of patients  treated by each care provider in each center) | Pages 11 & 12 |
|  | 13b | For each group, losses and exclusions after randomisation, together with reasons | Pages 11 & 12 |
| Recruitment | 14a | Dates defining the periods of recruitment and follow-up | Page 12 |
|  | 14b | Why the trial ended or was stopped | Page 12 |
| Baseline data | 15 | A table showing baseline demographic and clinical characteristics for each group (NPT: When applicable, a description of care providers (case volume, qualification, expertise, etc.) and centers (volume) in each group) | Pages 12 & 13 |
| Numbers analysed | 16 | For each group, number of participants (denominator) included in each analysis and whether the analysis was by original assigned groups | Pages 12 & 13 |
| Outcomes and estimation | 17a | For each primary and secondary outcome, results for each group, and the estimated effect size and its precision (such as 95% confidence interval) | Pages 13-18 |
|  | 17b | For binary outcomes, presentation of both absolute and relative effect sizes is recommended | Pages 13-18 |
| Ancillary analyses | 18 | Results of any other analyses performed, including subgroup analyses and adjusted analyses, distinguishing pre-specified from exploratory | Pages 18 |
| Harms | 19 | All important harms or unintended effects in each group (for specific guidance see CONSORT for harms) | Page 18 |
| Interpretation/Principle Findings | 22 | Interpretation consistent with results, balancing benefits and harms, and considering other relevant evidence (NPT: In addition, take into account the choice of the comparator, lack of or partial blinding, and unequal expertise of care providers or centers in each group) | Pages 13-18 |
| **Discussion** | | | |
| Limitations | 20 | Trial limitations, addressing sources of potential bias, imprecision, and, if relevant, multiplicity of analyses | Pages 19 & 20 |
| Generalisability | 21 | Generalisability (external validity, applicability) of the trial findings (NPT: External validity of the trial findings according to the intervention, comparators, patients, and care providers or centers involved in the trial) | Pages 18 & 19 |
| **Other information** | | |  |
| Registration | 23 | Registration number and name of trial registry | Page 3 |
| Protocol | 24 | Where the full trial protocol can be accessed, if available | Not applicable |
| Funding | 25 | Sources of funding and other support (such as supply of drugs), role of funders | Page 21 |
| Competing interests | X27 | (not a CONSORT item) | Page 21 |

*CONSORT, Consolidated Standards of Reporting Trials; NPT = non pharmacological treatment (CONSORT extension).

References

1. Baker TB, Gustafson DH, Shaw B, Hawkins R, Pingree S, Roberts L, Strecher V. Relevance of CONSORT reporting criteria for research on eHealth interventions. Patient Educ Couns. 2010 Dec;81 Suppl:S77-86

2. Talmon J, Ammenwerth E, Brender J, de Keizer N, Nykänen P, Rigby M. STARE-HI--Statement on reporting of evaluation studies in Health Informatics. Int J Med Inform. 2009 Jan;78(1):1-9. Epub 2008 Oct 18.

3. Eysenbach G. Issues in evaluating health websites in an Internet-based randomized controlled trial. J Med Internet Res 2002;4(3):e17

4. Blankers M, Koeter MWJ, Schippers GM. Missing Data Approaches in eHealth Research: Simulation Study and a Tutorial for Nonmathematically Inclined Researchers. J Med Internet Res 2010;12(5):e54

5. Eysenbach G. The law of attrition. J Med Internet Res 2005;7(1):e11

6. Proudfoot et al. Establishing Guidelines for Executing and Reporting Internet Intervention Research. Cognitive Behaviour Therapy (forthcoming)

7. Webb TL, Joseph J, Yardley L, Michie S. Using the Internet to Promote Health Behavior Change: A Systematic Review and Meta-analysis of the Impact of Theoretical Basis, Use of Behavior Change Techniques, and Mode of Delivery on Efficacy. J Med Internet Res 2010;12(1):e4

8. Cugelman B, Thelwall M, Dawes P. Online Interventions for Social Marketing Health Behavior Change Campaigns: A Meta-Analysis of Psychological Architectures and Adherence Factors. J Med Internet Res 2011;13(1):e17

9. Eysenbach G. Improving the Quality of Web Surveys: The Checklist for Reporting Results of Internet E-Surveys (CHERRIES). J Med Internet Res 2004;6(3):e34

10. Schulz KF, Altman DG, Moher D, for the CONSORT Group (2010) CONSORT 2010 Statement: Updated Guidelines for Reporting Parallel Group Randomised Trials. PLoS Med 7(3): e1000251

11. Boutron I, Moher D, Altman DG, Schulz K, Ravaud P, for the CONSORT group. Extending the CONSORT Statement to randomized trials of nonpharmacologic treatment: explanation and elaboration. Ann Intern Med. 2008:295-309
