# Supplementary material for: Efficacy of an Intelligent and Integrated Older Adult Care Model on Quality of Life Among Home-Dwelling Older Adults: Randomized Controlled Trial
Source: J Med Internet Res. 2025 Apr 21;27:e67950. doi: 10.2196/67950 (PMC12053148; doi:10.2196/67950)
Supplement: Multimedia Appendix 6 [file jmir_v27i1e67950_app6.docx]

**Efficacy of an Intelligent and Integrated Elderly Care Model on Quality of Life among Home-dwelling Elderly Adults: A Randomized Controlled Trial**

**CONTENTS**

**[Supplementary methods](#_Toc186398863)** [3](#_Toc186398863)

**[Supplementary imputation methods](#_Toc186398864)** [3](#_Toc186398864)

**[Table S1.](#_Toc186398865)** [The types and measurement methods of all covariates and endpoints along with the appropriate regression models for analysis 4](#_Toc186398865)

**[Supplementary results](#_Toc186398866)****[for the subgroup analysis by age group](#_Toc186398866)** [5](#_Toc186398866)

**[Table S2.](#_Toc186398867)** [Baseline characteristics of elderly participants in the intention-to-treat subgroup analysis by age group 5](#_Toc186398867)

**[Figure S1.](#_Toc186398868)** [Effects of the personalized and integrated care provided by the SMART system combined with usual care versus usual care on the primary and secondary endpoints in older adults aged 60-69 6](#_Toc186398868)

**[Figure S2.](#_Toc186398869)** [Effects of the personalized and integrated care provided by the SMART system combined with usual care versus usual care on the primary and secondary endpoints in older adults aged 70- 7](#_Toc186398869)

**[Table S3.](#_Toc186398870)** [Primary and secondary endpoints, along with the estimated differences observed among elderly participants aged 60-69 in the intention-to-treat analysis 8](#_Toc186398870)

**[Table S4.](#_Toc186398871)** [Primary and secondary endpoints, along with the estimated differences observed among elderly participants aged 70- in the intention-to-treat analysis 9](#_Toc186398871)

**[Supplementary results](#_Toc186398872)****[for the subgroup analysis by gender](#_Toc186398872)** [10](#_Toc186398872)

**[Table S5.](#_Toc186398873)** [Baseline characteristics of elderly participants in the intention-to-treat subgroup analysis by gender 10](#_Toc186398873)

**[Figure S3.](#_Toc186398874)** [Effects of the personalized and integrated care provided by the SMART system combined with usual care versus usual care on the primary and secondary endpoints in males 11](#_Toc186398874)

**[Figure S4.](#_Toc186398875)** [Effects of the personalized and integrated care provided by the SMART system combined with usual care versus usual care on the primary and secondary endpoints in females 12](#_Toc186398875)

**[Table S6.](#_Toc186398876)** [Primary and secondary endpoints, along with the estimated differences observed among males in the intention-to-treat analysis 13](#_Toc186398876)

**[Table S7.](#_Toc186398877)** [Primary and secondary endpoints, along with the estimated differences observed among females in the intention-to-treat analysis 14](#_Toc186398877)

**[Supplementary results](#_Toc186398878)****[for the subgroup analysis by BMI group](#_Toc186398878)** [15](#_Toc186398878)

**[Table S8.](#_Toc186398879)** [Baseline characteristics of elderly participants in the intention-to-treat subgroup analysis by BMI group 15](#_Toc186398879)

**[Figure S5.](#_Toc186398880)** [Effects of the personalized and integrated care provided by the SMART system combined with usual care versus usual care on the primary and secondary endpoints in older adults with normal BMI 16](#_Toc186398880)

**[Figure S6.](#_Toc186398881)** [Effects of the personalized and integrated care provided by the SMART system combined with usual care versus usual care on the primary and secondary endpoints in older adults with abnormal BMI 17](#_Toc186398881)

**[Table S9.](#_Toc186398882)** [Primary and secondary endpoints, along with the estimated differences observed among older adults with normal BMI in the intention-to-treat analysis 18](#_Toc186398882)

**[Table S10.](#_Toc186398883)** [Primary and secondary endpoints, along with the estimated differences observed among older adults with abnormal BMI in the intention-to-treat analysis 19](#_Toc186398883)

**[Supplementary results](#_Toc186398884)****[for the sensitivity analysis using relative change values as a measurement approach](#_Toc186398884)** [20](#_Toc186398884)

**[Table S11.](#_Toc186398885)** [Primary and secondary endpoints, along with the estimated differences observed in the intention-to-treat analysis when using relative change values as a measurement approach 20](#_Toc186398885)

**[Supplementary results](#_Toc186398886)****[of the per-protocol sensitivity analysis](#_Toc186398886)** [21](#_Toc186398886)

**[Table S12.](#_Toc186398887)** [Baseline characteristics of elderly participants in the per-protocol analysis 21](#_Toc186398887)

**[Figure S7.](#_Toc186398888)** [Effects of the personalized and integrated care provided by the SMART system combined with usual care versus usual care on the primary and secondary endpoints in the per-protocol analysis 22](#_Toc186398888)

**[Table S13.](#_Toc186398889)** [Primary and secondary endpoints, along with the estimated differences observed in the per-protocol analysis 23](#_Toc186398889)

**[References](#_Toc186398890)** [24](#_Toc186398890)

**Supplementary methods**

**Supplementary imputation methods**

Multiple Imputation by Chained Equations was performed to create a complete dataset since Little’s test suggested data were missing at random (*χ^2^*=13.8, *df*=12, *p*=.54). The number of iterations in each imputation was set at 100. The imputed 100 datasets were then each analyzed and the study results were pooled into the final study result using the “with” function of the mice package [1, 2, 3]. There is no missing value of the categorical variables in this study.

The imputed continuous variables and their respective missing rates in the current included: BMI (6, 6%), WHOQOL-OLD score (11, 12%), AHSMSRS score (13, 14%), SSRS score (11, 12%), ADL score (11, 12%), and MFES score (11, 12%).

Table S1. The types and measurement methods of all covariates and endpoints along with the appropriate regression models for analysis

| **Covariates/End points** | **Types** | **Measurement methods** | **Regression models** |
| --- | --- | --- | --- |
| **Covariates** |  |  |  |
| **Age** | Continuous variable | Continuous value | - |
| **Gender** | Categorical variable | Mage=1, Female=2 | - |
| **BMI (kg/m^2^)** | Continuous variable | Continuous value | - |
| **Marital status** | Categorical variable | Married=1,  Widowed=2,  Divorced=3 | - |
| **Education** | Categorical variable | Primary school and below=1,  Junior high school=2,  Senior high or vocational school=3,  College and above=4 | - |
| **Dwelling status** | Categorical variable | Living with others=1,  Living alone=2,  Living in a nursing home=3 | - |
| **Visual impairment** | Categorical variable | Yes=1, No=0 | - |
| **Hearing impairment** | Categorical variable | Yes=1, No=0 | - |
| **Smoking** | Categorical variable | Yes=1, No=0 | - |
| **Alcohol drinking** | Categorical variable | Yes=1, No=0 | - |
| **Primary end points** |  |  |  |
| **Percent change in WHOQOL-OLD scale** | Continuous variable | Continuous value | Covariance model |
| **Percent change in SAB score of the WHOQOL-OLD scale** | Continuous variable | Continuous value | Covariance model |
| **Percent change in AU score of the WHOQOL-OLD scale** | Continuous variable | Continuous value | Covariance model |
| **Percent change in DD score of the WHOQOL-OLD scale** | Continuous variable | Continuous value | Covariance model |
| **Percent change in PPFA score of the WHOQOL-OLD scale** | Continuous variable | Continuous value | Covariance model |
| **Percent change in SP score of the WHOQOL-OLD scale** | Continuous variable | Continuous value | Covariance model |
| **Percent change in INT score of the WHOQOL-OLD scale** | Continuous variable | Continuous value | Covariance model |
| **Secondary end points** |  |  |  |
| **ADL disability at the 3-month follow-up, No. (%)** | Categorical variable | Yes=1, No=0 | Logistic regression model |
| **IADL disability at the 3-month follow-up, No. (%)** | Categorical variable | Yes=1, No=0 | Logistic regression model |
| **Percent change in SSRS score** | Continuous variable | Continuous value | Covariance model |
| **Percent change in MFES score** | Continuous variable | Continuous value | Covariance model |
| **Percent change in ASHMAR score** | Continuous variable | Continuous value | Covariance model |

The table summarizes types and measurement methods of all covariates and endpoints, as well as the appropriate regression models to estimate the efficacy of different endpoints. BMI, body mass index; WHOQOL-OLD, World Health Organization Quality of Life Instrument-Older Adults Module; SAB, Sensory Abilities; AUT, Autonomy; DAD, Death and Dying; PPFA, Past, Present, and Future Activities; SP, Social Participation; INT, Intimacy; ADL, Activities of Daily Living; IADL, Instrumental Activities of Daily Living; SSRS, Social Support Rating Scale; MFES, Modified Fall Efficacy Scale; AHSMSRS, The Rating Scale of Health Self-Management Skill for Adults.

**Supplementary results** **for the subgroup analysis by age group**

Table S2. Baseline characteristics of older adults in the intention-to-treat subgroup analysis by age group (randomized controlled trial conducted from November 1 to December 31, 2020).

| **Variables** | **60≤Age≤69 (n=49)** | | | **Age≥70 (n=45)** | | |
| --- | --- | --- | --- | --- | --- | --- |
|  | **SMART**^a^ **group (n=28)** | **Usual care group (n=21)** | ***P value*** | **SMART**^a^ **group (n=20)** | **Usual care group (n=25)** | ***P value*** |
| **Age** | 66.00  (63.25, 67.75) | 66.00  (60.50, 68.00) | 0.855^b^ | 74.50  (71.25, 77.00) | 76.00  (71.00, 80.00) | 0.818^b^ |
| **Male** | 17 (60.71%) | 13 (61.90%) | 0.933^c^ | 12 (60.00%) | 15 (60.00%) | 0.999^c^ |
| **BMI^g^ (kg/m^2^)** | 24.38 ± 4.10 | 23.40 ± 2.77 | 0.351^b^ | 24.29 ± 3.64 | 23.20 ± 2.26 | 0.248^b^ |
| **Marital status** |  |  | 0.534^c^ |  |  | 0.205^c^ |
| **Married** | 21 (75.00%) | 18 (85.71%) |  | 11 (55.00%) | 19 (76.00%) |  |
| **Widowed** | 6 (21.43%) | 3 (14.29%) |  | 9 (45.00%) | 6 (24.00%) |  |
| **Divorced** | 1 (3.57%) | 0 (0.00%) |  | 0 (0.00%) | 0 (0.00%) |  |
| **Education** |  |  | 0.478^e^ |  |  | 0.981^e^ |
| **Primary school**  **and below** | 7 (25.00%) | 3 (14.29%) |  | 2 (10.00%) | 2 (8.00%) |  |
| **Junior high school** | 5 (17.86%) | 6 (28.57%) |  | 8 (40.00%) | 9 (36.00%) |  |
| **Senior high or**  **vocational school** | 10 (35.74%) | 5 (23.81%) |  | 7 (35.00%) | 10 (40.00%) |  |
| **College and above** | 6 (21.43%) | 7 (33.33%) |  | 3 (15.00%) | 4 (16.00%) |  |
| **Dwelling status** |  |  | -^d, f^ |  |  | 0.188^c^ |
| **Living with others** | 28 (100.00%) | 21 (100.00%) |  | 18 (90.00%) | 24 (96.00%) |  |
| **Living alone** | 0 (0.00%) | 0 (0.00%) |  | 2 (10.00%) | 0 (0.00%) |  |
| **Nursing home** | 0 (0.00%) | 0 (0.00%) |  | 0 (0.00%) | 1 (4.00%) |  |
| **Visual impairment** | 9 (32.14%) | 9 (42.86%) | 0.441^c^ | 10 (50.00%) | 8 (32.00%) | 0.221^c^ |
| **Hearing impairment** | 8 (28.57%) | 8 (38.10%) | 0.482^c^ | 9 (45.00%) | 8 (32.00%) | 0.371^c^ |
| **Smoking** | 8 (28.57%) | 2 (9.52%) | 0.102^c^ | 6 (30.00%) | 7 (28.00%) | 0.883^c^ |
| **Alcohol drinking** | 7 (25.00%) | 2 (9.52%) | 0.166^c^ | 3 (15.00%) | 4 (16.00%) | 0.927^c^ |

^a^SMART: Sensors and scales (receptor), a Mobile phone autonomous response system (central nervous system in the spinal cord), a Remote cloud management center (central nervous system in the brain), and a Total care system (effector).

^b^Student t test.

^c^chi-square test.

^d^Fisher exact test.

^e^Wilcoxon rank-sum test.

^f^not applicable.

^g^BMI, body mass index.

Figure S1. Effects of the personalized and integrated care provided by the SMART system combined with usual care versus usual care on the primary and secondary endpoints in older adults aged 60-69. (A) Effects of the personalized and integrated care provided by the SMART system combined with usual care versus usual care on quality of life in older adults aged 60-69. (B) Effects of the personalized and integrated care provided by the SMART system combined with usual care versus usual care on functional status in older adults aged 60-69. (C) Effects of the personalized and integrated care provided by the SMART system combined with usual care versus usual care on other secondary outcomes in older adults aged 60-69. WHOQOL-OLD, World Health Organization Quality of Life Instrument-Older Adults Module; SAB, Sensory Abilities; AUT, Autonomy; DAD, Death and Dying; PPFA, Past, Present, and Future Activities; SP, Social Participation; INT, Intimacy; ADL, Activities of Daily Living; IADL, Instrumental Activities of Daily Living; SSRS, Social Support Rating Scale; MFES, Modified Fall Efficacy Scale; AHSMSRS, The Rating Scale of Health Self-Management Skill for Adults.

Figure S2. Effects of the personalized and integrated care provided by the SMART system combined with usual care versus usual care on the primary and secondary endpoints in older adults aged 70-. (A) Effects of the personalized and integrated care provided by the SMART system combined with usual care versus usual care on quality of life in older adults aged 70-. (B) Effects of the personalized and integrated care provided by the SMART system combined with usual care versus usual care on functional status in older adults aged 70-. (C) Effects of the personalized and integrated care provided by the SMART system combined with usual care versus usual care on other secondary outcomes in older adults aged 70-. WHOQOL-OLD, World Health Organization Quality of Life Instrument-Older Adults Module; SAB, Sensory Abilities; AUT, Autonomy; DAD, Death and Dying; PPFA, Past, Present, and Future Activities; SP, Social Participation; INT, Intimacy; ADL, Activities of Daily Living; IADL, Instrumental Activities of Daily Living; SSRS, Social Support Rating Scale; MFES, Modified Fall Efficacy Scale; AHSMSRS, The Rating Scale of Health Self-Management Skill for Adults.

Table S3. Primary and secondary endpoints, along with the estimated differences observed among elderly participants aged 60-69 in the intention-to-treat analysis

| **Endpoints** | **SMART**^a^ **group (n=28)** | **Usual care group (n=21)** | **Difference**^c^ **(95% CI)^a^** | **OR**^d^ **(95%CI)** | ***P value*** |
| --- | --- | --- | --- | --- | --- |
| **Primary endpoints** |  |  |  |  |  |
| **Percent change**^b^ **in WHOQOL-OLD**^e^ **score** | 25.93  (21.55, 30.31) | 16.49  (12.91, 20.07) | 9.44  (3.93, 14.95) | -^f^ | <0.001^g^ |
| **Percent change**^b^ **in SAB**^h^ **score of the WHOQOL-OLD scale** | 86.92  (74.02, 99.82) | 43.09  (34.07, 52.11) | 43.83  (28.48, 59.18) | -^f^ | <0.001^g^ |
| **Percent change**^b^ **in AUT**^i^ **score of the WHOQOL-OLD scale** | 14.36  (8.72, 19.99) | 6.92  (1.45, 12.39) | 7.44  (2.03, 15.08) | -^f^ | 0.007^g^ |
| **Percent change**^b^ **in DAD**^j^ **score of the WHOQOL-OLD scale** | 36.11  (19.07, 53.16) | 34.74  (9.46, 60.02) | 1.37  (-28.18, 30.93) | -^f^ | 0.823 |
| **Percent change**^b^ **in PPFA**^k^ **score of the WHOQOL-OLD scale** | 16.55  (9.83, 23.28) | 8.67  (2.87, 14.48) | 7.88  (0.77, 16.53) | -^f^ | 0.012^g^ |
| **Percent change**^b^ **in SP**^l^ **score of the WHOQOL-OLD scale** | 19.80  (12.30, 27.30) | 9.17  (3.78, 14.56) | 10.63  (1.62, 19.63) | -^f^ | <0.001^g^ |
| **Percent change**^b^ **in INT**^m^ **score of the WHOQOL-OLD scale** | 28.63  (15.72, 41.53) | 35.39  (15.94, 54.84) | -6.76  (-29.38, 15.87) | -^f^ | 0.251 |
| **Secondary endpoints** |  |  |  |  |  |
| **Participants with ADL**^n^ **disability at 3-month follow-up, No. (%)** | 14 (50.00%) | 13 (61.90%) | -11.90  (-40.48, 16.66) | 1.28  (0.18, 10.86) | 0.805 |
| **Participants with IADL**^o^ **disability at 3-month follow-up, No. (%)** | 12 (42.86%) | 13 (61.90%) | -19.05  (-47.48, 9.39) | 0.42  (0.12, 1.37) | 0.158 |
| **Percent change**^b^ **in SSRS**^p^ **score** | 6.43  (3.38, 9.49) | 1.21  (0.03, 2.39) | 5.22  (2.02, 8.43) | -^f^ | 0.001^g^ |
| **Percent change**^b^ **in MFES**^q^ **score** | 5.12  (2.24, 8.02) | 4.89  (1.54, 8.23) | 0.24  (-4.06, 4.54) | -^f^ | 0.821 |
| **Percent change**^b^ **in ASHMAR**^r^ **score** | 9.87  (4.84, 14.91) | 5.96  (0.35, 11.57) | 3.91  (-3.41, 11.24) | -^f^ | 0.135 |

^a^SMART: Sensors and scales (receptor), a Mobile phone autonomous response system (central nervous system in the spinal cord), a Remote cloud management center (central nervous system in the brain), and a Total care system (effector).

^b^The percent change values are presented as “mean (95% CI)” values.

^c^Data are absolute differences between mean changes and expressed in percentage points.

^d^OR: odds ratio.

^e^WHOQOL-OLD: World Health Organization Quality of Life Instrument-Older Adults Module.

^f^Not applicable

^g^Indicate statistically significant variables (*P*<.05).

^h^SAB: sensory abilities.

^i^AUT: autonomy.

^j^DAD: death and dying.

^k^PPFA: past, present, and future activities.

^l^SP: social participation.

^m^INT: intimacy.

^n^ADL: activities of daily living.

^o^IADL: instrumental activities of daily living

^p^SSRS: Social Support Rating Scale.

^q^MFES: Modified Fall Efficacy Scale.

^r^AHSMSRS: The Rating Scale of Health Self-Management Skill for Adults.

Table S4. Primary and secondary endpoints, along with the estimated differences observed among elderly participants aged 70- in the intention-to-treat analysis

| **Endpoints** | **SMART**^a^ **group (n=20)** | **Usual care group (n=25)** | **Difference**^c^ **(95% CI)^a^** | **OR**^d^ **(95%CI)** | ***P value*** |
| --- | --- | --- | --- | --- | --- |
| **Primary endpoints** |  |  |  |  |  |
| **Percent change**^b^ **in WHOQOL-OLD**^e^ **score** | 34.63  (28.46, 40.80) | 18.51  (13.43, 23.59) | 16.12  (8.37, 23.87) | -^f^ | <0.001^g^ |
| **Percent change**^b^ **in SAB**^h^ **score of the WHOQOL-OLD scale** | 65.78  (47.04, 84.53) | 38.24  (23.13, 53.35) | 27.54  (4.21, 50.87) | -^f^ | <0.001^g^ |
| **Percent change**^b^ **in AUT**^i^ **score of the WHOQOL-OLD scale** | 29.06  (16.74, 41.38) | 13.89  (6.62, 21.16) | 15.17  (1.33, 29.00) | -^f^ | <0.001^g^ |
| **Percent change**^b^ **in DAD**^j^ **score of the WHOQOL-OLD scale** | 46.66  (21.05, 72.28) | 40.63  (14.45, 66.81) | 6.03  (-29.51, 41.58) | -^f^ | 0.419 |
| **Percent change**^b^ **in PPFA**^k^ **score of the WHOQOL-OLD scale** | 24.95  (14.99, 34.91) | 12.45  (6.29, 18.61) | 12.50  (1.17, 23.82) | -^f^ | 0.004^g^ |
| **Percent change**^b^ **in SP**^l^ **score of the WHOQOL-OLD scale** | 36.92  (23.65, 50.18) | 17.01  (10.09, 23.84) | 19.90  (5.49, 34.32) | -^f^ | <0.001^g^ |
| **Percent change**^b^ **in INT**^m^ **score of the WHOQOL-OLD scale** | 43.27  (24.42, 62.12) | 30.27  (12.42, 48.13) | 13.00  (-12.19, 38.18 ) | -^f^ | 0.067 |
| **Secondary endpoints** |  |  |  |  |  |
| **Participants with ADL**^n^ **disability at 3-month follow-up, No. (%)** | 4 (20.00%) | 9 (36.00%) | -16.00  (-42.46, 10.46) | 0.13  (0.01, 1.11) | 0.096 |
| **Participants with IADL**^o^ **disability at 3-month follow-up, No. (%)** | 15 (75.00%) | 17 (68.00%) | -15.00  (-44.89, -4.90) | 0.09  (0.01, 0.77) | 0.046^g^ |
| **Percent change**^b^ **in SSRS**^p^ **score** | 7.64  (1.43, 13.86) | -0.66  (-4.28, 2.95) | 8.31  (1.35, 15.26) | -^f^ | 0.003^g^ |
| **Percent change**^b^ **in MFES**^q^ **score** | 12.97  (5.18, 20.75) | 6.04  (2.32, 9.76) | 6.93  (1.41, 15.26) | -^f^ | <0.001^g^ |
| **Percent change**^b^ **in ASHMAR**^r^ **score** | 11.63  (6.71, 16.55) | 5.40  (-0.09, 10.89) | 6.22  (0.94, 13.38) | -^f^ | 0.004^g^ |

^a^SMART: Sensors and scales (receptor), a Mobile phone autonomous response system (central nervous system in the spinal cord), a Remote cloud management center (central nervous system in the brain), and a Total care system (effector).

^b^The percent change values are presented as “mean (95% CI)” values.

^c^Data are absolute differences between mean changes and expressed in percentage points.

^d^OR: odds ratio.

^e^WHOQOL-OLD: World Health Organization Quality of Life Instrument-Older Adults Module.

^f^Not applicable

^g^Indicate statistically significant variables (*P*<.05).

^h^SAB: sensory abilities.

^i^AUT: autonomy.

^j^DAD: death and dying.

^k^PPFA: past, present, and future activities.

^l^SP: social participation.

^m^INT: intimacy.

^n^ADL: activities of daily living.

^o^IADL: instrumental activities of daily living

^p^SSRS: Social Support Rating Scale.

^q^MFES: Modified Fall Efficacy Scale.

^r^AHSMSRS: The Rating Scale of Health Self-Management Skill for Adults.

**Supplementary results** **for the subgroup analysis by gender**

Table S5. Baseline characteristics of elderly participants in the intention-to-treat subgroup analysis by gender (randomized controlled trial conducted from November 1 to December 31, 2020).

| **Variables** | **Male (n=57)** | | | **Female (n=37)** | | |
| --- | --- | --- | --- | --- | --- | --- |
|  | **SMART**^a^ **group (n=29)** | **Usual care group (n=28)** | ***P value*** | **SMART**^a^ **group (n=19)** | **Usual care group (n=18)** | ***P value*** |
| **Age** | 68.66 ± 5.58 | 71.11 ± 7.27 | 0.158^b^ | 70.79 ± 7.75 | 70.39 ± 7.03 | 0.870^b^ |
| **BMI (kg/m^2^)** | 24.04 ± 3.60 | 23.32 ±2.49 | 0.385^b^ | 24.81 ± 4.32 | 23.24 ± 2.53 | 0.189^b^ |
| **Marital status** |  |  | 0.495^c^ |  |  | 0.197^c^ |
| **Married** | 21 (72.41%) | 23 (82.14%) |  | 11 (57.89%) | 14 (77.78%) |  |
| **Widowed** | 7 (24.14%) | 5 (17.86%) |  | 8 (42.11%) | 4 (22.22%) |  |
| **Divorced** | 1 (3.45%) | 0 (0.00%) |  | 0 (0.00%) | 0 (0.00%) |  |
| **Education** |  |  | 0.484^e^ |  |  | 0.227^e^ |
| **Primary school**  **and below** | 4 (13.79%) | 4 (14.29%) |  | 5 (26.32%) | 1 (5.56%) |  |
| **Junior high school** | 8 (27.59%) | 12 (42.86%) |  | 5 (26.32%) | 3 (16.67%) |  |
| **Senior high or**  **vocational school** | 10 (3.45%) | 5 (17.86%) |  | 7 (36.84%) | 10 (55.56%) |  |
| **College and above** | 7 (24.14%) | 7 (25.00%) |  | 2 (10.53%) | 4 (22.22%) |  |
| **Dwelling status** |  |  | -^d, f^ |  |  | 0.226^c^ |
| **Living with others** | 29 (100.00%) | 28 (100.00%) |  | 17 (89.47%) | 17 (94.44%) |  |
| **Living alone** | 0 (0.00%) | 0 (0.00%) |  | 2 (10.53%) | 0 (0.00%) |  |
| **Nursing home** | 0 (0.00%) | 0 (0.00%) |  | 0 (0.00%) | 1 (5.56%) |  |
| **Visual impairment** | 11 (37.93%) | 12 (42.86%) | 0.705^c^ | 8 (42.11%) | 5 (27.78%) | 0.362^c^ |
| **Hearing impairment** | 11 (37.93%) | 9 (32.14%) | 0.647^c^ | 6 (31.58%) | 7 (38.89%) | 0.642^c^ |
| **Smoking** | 9 (31.03%) | 5 (17.86%) | 0.248^c^ | 5 (26.32%) | 4 (22.22%) | 0.772^c^ |
| **Alcohol drinking** | 5 (17.24%) | 3 (10.71%) | 0.478^c^ | 5 (26.32%) | 3 (16.67%) | 0.476^c^ |

^a^SMART: Sensors and scales (receptor), a Mobile phone autonomous response system (central nervous system in the spinal cord), a Remote cloud management center (central nervous system in the brain), and a Total care system (effector).

^b^Student t test.

^c^chi-square test.

^d^Fisher exact test.

^e^Wilcoxon rank-sum test.

^f^not applicable.

^g^BMI, body mass index.

Figure S3. Effects of the personalized and integrated care provided by the SMART system combined with usual care versus usual care on the primary and secondary endpoints in males. (A) Effects of the personalized and integrated care provided by the SMART system combined with usual care versus usual care on quality of life in males. (B) Effects of the personalized and integrated care provided by the SMART system combined with usual care versus usual care on functional status in males. (C) Effects of the personalized and integrated care provided by the SMART system combined with usual care versus usual care on other secondary outcomes in males. WHOQOL-OLD, World Health Organization Quality of Life Instrument-Older Adults Module; SAB, Sensory Abilities; AUT, Autonomy; DAD, Death and Dying; PPFA, Past, Present, and Future Activities; SP, Social Participation; INT, Intimacy; ADL, Activities of Daily Living; IADL, Instrumental Activities of Daily Living; SSRS, Social Support Rating Scale; MFES, Modified Fall Efficacy Scale; AHSMSRS, The Rating Scale of Health Self-Management Skill for Adults.

Figure S4. Effects of the personalized and integrated care provided by the SMART system combined with usual care versus usual care on the primary and secondary endpoints in females. (A) Effects of the personalized and integrated care provided by the SMART system combined with usual care versus usual care on quality of life in females. (B) Effects of the personalized and integrated care provided by the SMART system combined with usual care versus usual care on functional status in females. (C) Effects of the personalized and integrated care provided by the SMART system combined with usual care versus usual care on other secondary outcomes in females. WHOQOL-OLD, World Health Organization Quality of Life Instrument-Older Adults Module; SAB, Sensory Abilities; AUT, Autonomy; DAD, Death and Dying; PPFA, Past, Present, and Future Activities; SP, Social Participation; INT, Intimacy; ADL, Activities of Daily Living; IADL, Instrumental Activities of Daily Living; SSRS, Social Support Rating Scale; MFES, Modified Fall Efficacy Scale; AHSMSRS, The Rating Scale of Health Self-Management Skill for Adults.

Table S6. Primary and secondary endpoints, along with the estimated differences observed among males in the intention-to-treat analysis

| **Endpoints** | **SMART**^a^ **group (n=29)** | **Usual care group (n=28)** | **Difference**^c^ **(95% CI)^a^** | **OR**^d^ **(95%CI)** | ***P value*** |
| --- | --- | --- | --- | --- | --- |
| **Primary endpoints** |  |  |  |  |  |
| **Percent change**^b^ **in WHOQOL-OLD**^e^ **score** | 31.18  (27.16, 35.20) | 19.32  (15.14, 23.50) | 11.86  (6.19, 17.53) | -^f^ | <0.001^g^ |
| **Percent change**^b^ **in SAB**^h^ **score of the WHOQOL-OLD scale** | 84.46  (68.72, 99.90) | 45.15  (32.81, 57.49) | 39.31  (19.75, 58.87) | -^f^ | <0.001^g^ |
| **Percent change**^b^ **in AUT**^i^ **score of the WHOQOL-OLD scale** | 18.11  (9.29, 26.94) | 9.80  (3.34, 16.26) | 8.31  (2.38, 19.00) | -^f^ | 0.012^g^ |
| **Percent change**^b^ **in DAD**^j^ **score of the WHOQOL-OLD scale** | 52.52  (31.78, 73.27) | 51.84  (27.27, 76.40) | 0.68  (-30.74, 32.11) | -^f^ | 0.914 |
| **Percent change**^b^ **in PPFA**^k^ **score of the WHOQOL-OLD scale** | 19.49  (13.38, 25.60) | 11.89  (6.39, 17.40) | 7.60  (1.44, 15.63) | -^f^ | 0.017^g^ |
| **Percent change**^b^ **in SP**^l^ **score of the WHOQOL-OLD scale** | 24.61  (16.14, 33.07) | 13.74  (8.05, 19.42) | 10.87  (6.90, 20.84) | -^f^ | <0.001^g^ |
| **Percent change**^b^ **in INT**^m^ **score of the WHOQOL-OLD scale** | 40.37  (25.64, 55.10) | 25.46  (12.89, 38.03) | 14.91  (4.02, 33.84) | -^f^ | 0.004^g^ |
| **Secondary endpoints** |  |  |  |  |  |
| **Participants with ADL**^n^ **disability at 3-month follow-up, No. (%)** | 10 (34.48%) | 13 (46.29%) | -11.94  (-37.82, 13.93) | 0.15  (0.01, 1.16) | 0.111 |
| **Participants with IADL**^o^ **disability at 3-month follow-up, No. (%)** | 11 (37.93%) | 18 (64.29%) | -26.35  (-51.95, -0.75) | 0.29  (0.08, 0.97) | 0.046^g^ |
| **Percent change**^b^ **in SSRS**^p^ **score** | 5.04  (3.08, 7.00) | 1.35  (-0.07, 2.78) | 3.69  (1.32, 6.06) | -^f^ | <0.001^g^ |
| **Percent change**^b^ **in MFES**^q^ **score** | 4.03  (1.51, 6.56) | 4.97  (1.66, 8.29) | -0.94  (-5.01, 3.13) | -^f^ | 0.334 |
| **Percent change**^b^ **in ASHMAR**^r^ **score** | 10.29  (5.82, 14.75) | 5.76  (1.62, 9.90) | 4.53  (1.43, 10.48) | -^f^ | 0.042^g^ |

^a^SMART: Sensors and scales (receptor), a Mobile phone autonomous response system (central nervous system in the spinal cord), a Remote cloud management center (central nervous system in the brain), and a Total care system (effector).

^b^The percent change values are presented as “mean (95% CI)” values.

^c^Data are absolute differences between mean changes and expressed in percentage points.

^d^OR: odds ratio.

^e^WHOQOL-OLD: World Health Organization Quality of Life Instrument-Older Adults Module.

^f^Not applicable

^g^Indicate statistically significant variables (*P*<.05).

^h^SAB: sensory abilities.

^i^AUT: autonomy.

^j^DAD: death and dying.

^k^PPFA: past, present, and future activities.

^l^SP: social participation.

^m^INT: intimacy.

^n^ADL: activities of daily living.

^o^IADL: instrumental activities of daily living

^p^SSRS: Social Support Rating Scale.

^q^MFES: Modified Fall Efficacy Scale.

^r^AHSMSRS: The Rating Scale of Health Self-Management Skill for Adults.

Table S7. Primary and secondary endpoints, along with the estimated differences observed among females in the intention-to-treat analysis

| **Endpoints** | **SMART**^a^ **group (n=19)** | **Usual care group (n=18)** | **Difference**^c^ **(95% CI)^a^** | **OR**^d^ **(95%CI)** | ***P value*** |
| --- | --- | --- | --- | --- | --- |
| **Primary endpoints** |  |  |  |  |  |
| **Percent change**^b^ **in WHOQOL-OLD**^e^ **score** | 27.08  (19.61, 34.54) | 14.90  (10.13, 19.66) | 12.18  (3.63, 20.73) | -^f^ | <0.001^g^ |
| **Percent change**^b^ **in SAB**^h^ **score of the WHOQOL-OLD scale** | 68.42  (54.66, 82.18) | 33.15  (20.30, 46.00) | 35.27  (17.11, 53.43) | -^f^ | <0.001^g^ |
| **Percent change**^b^ **in AUT**^i^ **score of the WHOQOL-OLD scale** | 24.10  (15.36, 32.83) | 12.12  (5.15, 19.09) | 11.98  (1.19, 22.76) | -^f^ | 0.001^g^ |
| **Percent change**^b^ **in DAD**^j^ **score of the WHOQOL-OLD scale** | 22.17  (7.57, 36.77) | 16.32  (-6.69, 39.33) | 5.85  (-20.41, 32.10) | -^f^ | 0.248 |
| **Percent change**^b^ **in PPFA**^k^ **score of the WHOQOL-OLD scale** | 20.91  (9.38, 32.44) | 8.91  (2.07, 15.76) | 12.00  (0.95, 24.94) | -^f^ | 0.004^g^ |
| **Percent change**^b^ **in SP**^l^ **score of the WHOQOL-OLD scale** | 30.47  (16.79, 44.16) | 12.96  (5.09, 20.83) | 17.52  (2.28, 32.75) | -^f^ | <0.001^g^ |
| **Percent change**^b^ **in INT**^m^ **score of the WHOQOL-OLD scale** | 26.12  (10.50, 41.74) | 43.73  (16.84, 70.61) | -17.61  (-47.56, 12.34) | -^f^ | 0.078 |
| **Secondary endpoints** |  |  |  |  |  |
| **Participants with ADL**^n^ **disability at 3-month follow-up, No. (%)** | 8 (42.11%) | 9 (50.00%) | -7.89  (-41.08, 25.29) | 2.40  (0.26, 53.30) | 0.477 |
| **Participants with IADL**^o^ **disability at 3-month follow-up, No. (%)** | 10 (52.63%) | 10 (55.56%) | -2.92  (-36.18, 30.33) | 0.36  (0.06, 1.71) | 0.219 |
| **Percent change**^b^ **in SSRS**^p^ **score** | 9.94  (2.62, 17.05) | -1.61  (-6.37, 3.14) | 11.45  (3.11, 19.79) | -^f^ | <0.001^g^ |
| **Percent change**^b^ **in MFES**^q^ **score** | 15.04  (7.12, 22.97) | 6.35  (2.45, 10.26) | 8.69  (0.16, 17.22) | -^f^ | <0.001^g^ |
| **Percent change**^b^ **in ASHMAR**^r^ **score** | 11.09  (5.05, 17.13) | 5.50  (-2.32, 13.32) | 5.59  (3.94, 15.11) | -^f^ | 0.017^g^ |

^a^SMART: Sensors and scales (receptor), a Mobile phone autonomous response system (central nervous system in the spinal cord), a Remote cloud management center (central nervous system in the brain), and a Total care system (effector).

^b^The percent change values are presented as “mean (95% CI)” values.

^c^Data are absolute differences between mean changes and expressed in percentage points.

^d^OR: odds ratio.

^e^WHOQOL-OLD: World Health Organization Quality of Life Instrument-Older Adults Module.

^f^Not applicable

^g^Indicate statistically significant variables (*P*<.05).

^h^SAB: sensory abilities.

^i^AUT: autonomy.

^j^DAD: death and dying.

^k^PPFA: past, present, and future activities.

^l^SP: social participation.

^m^INT: intimacy.

^n^ADL: activities of daily living.

^o^IADL: instrumental activities of daily living

^p^SSRS: Social Support Rating Scale.

^q^MFES: Modified Fall Efficacy Scale.

^r^AHSMSRS: The Rating Scale of Health Self-Management Skill for Adults.

**Supplementary results** **for the subgroup analysis by BMI group**

Table S8. Baseline characteristics of elderly participants in the intention-to-treat subgroup analysis by BMI group (randomized controlled trial conducted from November 1 to December 31, 2020).

| **Variables** | **Normal BMI (n=48)** | | | **Abnormal BMI (n=46)** | | |
| --- | --- | --- | --- | --- | --- | --- |
|  | **SMART**^a^ **group (n=21)** | **Usual care group (n=27)** | ***P value*** | **SMART**^a^ **group (n=27)** | **Usual care group (n=19)** | ***P value*** |
| **Age** | 68.57 ± 6.82 | 71.52 ± 7.94 | 0.182^b^ | 70.22 ± 6.33 | 69.84 ± 5.79 | 0.837^b^ |
| **Male** | 13 (61.90%) | 17 (62.96%) | 0.940^c^ | 16 (59.26%) | 11 (57.89%) | 0.926^c^ |
| **BMI**^e^ **(kg/m^2^)** | 21.78  (21.12, 23.25) | 21.80  (20.98, 22.77) | 0.701^b^ | 26.34  (25.30, 28.49) | 25.41  (24.65, 27.29) | 0.101^b^ |
| **Marital status** |  |  | 0.614^c^ |  |  | 0.256^c^ |
| **Married** | 15 (71.43%) | 21 (77.78%) |  | 17 (62.96%) | 16 (84.21%) |  |
| **Widowed** | 6 (28.57%) | 6 (22.22%) |  | 9 (33.33%) | 3 (15.79%) |  |
| **Divorced** | 0 (0.00%) | 0 (0.00%) |  | 1 (3.70%) | 0 (0.00%) |  |
| **Education** |  |  | 0.985^d^ |  |  | 0.646^d^ |
| **Primary school**  **and below** | 3 (14.29%) | 3 (11.11%) |  | 6 (22.22%) | 2 (10.53%) |  |
| **Junior high school** | 7 (33.33%) | 9 (33.33%) |  | 6 (22.22%) | 6 (31.58%) |  |
| **Senior high or**  **vocational school** | 7 (33.33%) | 9 (33.33%) |  | 10 (37.04%) | 6 (31.58%) |  |
| **College and above** | 4 (19.05%) | 6 (22.22%) |  | 5 (18.52%) | 5 (26.32%) |  |
| **Dwelling status** |  |  | 0.373^c^ |  |  | 0.225^c^ |
| **Living with others** | 21 (100.00%) | 26 (96.30%) |  | 25 (92.59%) | 19 (100.00%) |  |
| **Living alone** | 0 (0.00%) | 0 (0.00%) |  | 2 (7.41%) | 0 (0.00%) |  |
| **Nursing home** | 0 (0.00%) | 1 (3.70%) |  | 0 (0.00%) | 0 (0.00%) |  |
| **Visual impairment** | 9 (42.86%) | 11 (40.74%) | 0.883^c^ | 10 (37.04%) | 6 (31.58%) | 0.702^c^ |
| **Hearing impairment** | 10 (47.62%) | 9 (33.33%) | 0.315^c^ | 7 (25.93%) | 7 (36.84%) | 0.428^c^ |
| **Smoking** | 6 (28.57%) | 5 (18.52%) | 0.411^c^ | 8 (29.63%) | 4 (21.05%) | 0.514^c^ |
| **Alcohol drinking** | 5 (23.81%) | 0 (0.00%) | 0.028^c^ | 5 (18.52%) | 6 (31.58%) | 0.307^c^ |

^a^SMART: Sensors and scales (receptor), a Mobile phone autonomous response system (central nervous system in the spinal cord), a Remote cloud management center (central nervous system in the brain), and a Total care system (effector).

^b^Student t test.

^c^chi-square test.

^d^Wilcoxon rank-sum test.

^e^BMI, body mass index.

Figure S5. Effects of the personalized and integrated care provided by the SMART system combined with usual care versus usual care on the primary and secondary endpoints in older adults with normal BMI. (A) Effects of the personalized and integrated care provided by the SMART system combined with usual care versus usual care on quality of life in older adults with normal BMI. (B) Effects of the personalized and integrated care provided by the SMART system combined with usual care versus usual care on functional status in older adults with normal BMI. (C) Effects of the personalized and integrated care provided by the SMART system combined with usual care versus usual care on other secondary outcomes in older adults with normal BMI. WHOQOL-OLD, World Health Organization Quality of Life Instrument-Older Adults Module; SAB, Sensory Abilities; AUT, Autonomy; DAD, Death and Dying; PPFA, Past, Present, and Future Activities; SP, Social Participation; INT, Intimacy; ADL, Activities of Daily Living; IADL, Instrumental Activities of Daily Living; SSRS, Social Support Rating Scale; MFES, Modified Fall Efficacy Scale; AHSMSRS, The Rating Scale of Health Self-Management Skill for Adults.

Figure S6. Effects of the personalized and integrated care provided by the SMART system combined with usual care versus usual care on the primary and secondary endpoints in older adults with abnormal BMI. (A) Effects of the personalized and integrated care provided by the SMART system combined with usual care versus usual care on quality of life in older adults with abnormal BMI. (B) Effects of the personalized and integrated care provided by the SMART system combined with usual care versus usual care on functional status in older adults with abnormal BMI. (C) Effects of the personalized and integrated care provided by the SMART system combined with usual care versus usual care on other secondary outcomes in older adults with abnormal BMI. WHOQOL-OLD, World Health Organization Quality of Life Instrument-Older Adults Module; SAB, Sensory Abilities; AUT, Autonomy; DAD, Death and Dying; PPFA, Past, Present, and Future Activities; SP, Social Participation; INT, Intimacy; ADL, Activities of Daily Living; IADL, Instrumental Activities of Daily Living; SSRS, Social Support Rating Scale; MFES, Modified Fall Efficacy Scale; AHSMSRS, The Rating Scale of Health Self-Management Skill for Adults.

Table S9. Primary and secondary endpoints, along with the estimated differences observed among older adults with normal BMI in the intention-to-treat analysis

| **Endpoints** | **SMART**^a^ **group (n=21)** | **Usual care group (n=27)** | **Difference**^c^ **(95% CI)^a^** | **OR**^d^ **(95%CI)** | ***P value*** |
| --- | --- | --- | --- | --- | --- |
| **Primary endpoints** |  |  |  |  |  |
| **Percent change**^b^ **in WHOQOL-OLD**^e^ **score** | 25.93  (20.04, 31.82) | 19.52  (14.96, 24.07) | 6.41  (0.81, 13.64) | -^f^ | 0.005^g^ |
| **Percent change**^b^ **in SAB**^h^ **score of the WHOQOL-OLD scale** | 68.26  (52.47, 84.05) | 43.46  (31.32, 55.60) | 24.80  (5.47, 44.13) | -^f^ | <0.001^g^ |
| **Percent change**^b^ **in AUT**^i^ **score of the WHOQOL-OLD scale** | 16.09  (8.74, 23.44) | 14.92  (8.51, 21.32) | 1.17  (-8.30, 10.64) | -^f^ | 0.678 |
| **Percent change**^b^ **in DAD**^j^ **score of the WHOQOL-OLD scale** | 30.58  (8.83, 52.34) | 38.11  (15.89, 60.32) | -7.52  (-37.76, 22.71) | -^f^ | 0.243 |
| **Percent change**^b^ **in PPFA**^k^ **score of the WHOQOL-OLD scale** | 15.32  (10.18, 20.46) | 13.34  (7.12, 19.55) | 1.98  (-5.87, 9.84) | -^f^ | 0.488 |
| **Percent change**^b^ **in SP**^l^ **score of the WHOQOL-OLD scale** | 28.58  (16.26, 40.89) | 15.77  (9.19, 22.36) | 12.81  (1.72, 26.33) | -^f^ | <0.001^g^ |
| **Percent change**^b^ **in INT**^m^ **score of the WHOQOL-OLD scale** | 35.50  (17.83, 53.17) | 28.01  (13.72, 42.31) | 7.49  (-14.57, 29.55) | -^f^ | 0.217 |
| **Secondary endpoints** |  |  |  |  |  |
| **Participants with ADL**^n^ **disability at 3-month follow-up, No. (%)** | 8 (38.10%) | 13 (48.15%) | -10.05  (-38.86, 18.75) | 1.07  (0.08, 2.55) | 0.960 |
| **Participants with IADL**^o^ **disability at 3-month follow-up, No. (%)** | 10 (47.62%) | 16 (59.26%) | -11.64  (-40.68, 17.40) | 0.45  (0.09,1.92) | 0.290 |
| **Percent change**^b^ **in SSRS**^p^ **score** | 6.41  (2.84, 9.97) | 0.58  (-1.67, 2.81) | 5.83  (1.75, 9.91) | -^f^ | <0.001^g^ |
| **Percent change**^b^ **in MFES**^q^ **score** | 8.11  (3.25, 12.97) | 3.70  (0.67, 6.72) | 4.42  (-1.13, 9.97) | -^f^ | 0.343 |
| **Percent change**^b^ **in ASHMAR**^r^ **score** | 8.95  (3.70, 14.19) | 6.67  (2.06, 11.28) | 2.27  (1.51, 9.05) | -^f^ | <0.001^g^ |

^a^SMART: Sensors and scales (receptor), a Mobile phone autonomous response system (central nervous system in the spinal cord), a Remote cloud management center (central nervous system in the brain), and a Total care system (effector).

^b^The percent change values are presented as “mean (95% CI)” values.

^c^Data are absolute differences between mean changes and expressed in percentage points.

^d^OR: odds ratio.

^e^WHOQOL-OLD: World Health Organization Quality of Life Instrument-Older Adults Module.

^f^Not applicable

^g^Indicate statistically significant variables (*P*<.05).

^h^SAB: sensory abilities.

^i^AUT: autonomy.

^j^DAD: death and dying.

^k^PPFA: past, present, and future activities.

^l^SP: social participation.

^m^INT: intimacy.

^n^ADL: activities of daily living.

^o^IADL: instrumental activities of daily living

^p^SSRS: Social Support Rating Scale.

^q^MFES: Modified Fall Efficacy Scale.

^r^AHSMSRS: The Rating Scale of Health Self-Management Skill for Adults.

Table S10. Primary and secondary endpoints, along with the estimated differences observed among older adults with abnormal BMI in the intention-to-treat analysis

| **Endpoints** | **SMART**^a^ **group (n=27)** | **Usual care group (n=19)** | **Difference**^c^ **(95% CI)^a^** | **OR**^d^ **(95%CI)** | ***P value*** |
| --- | --- | --- | --- | --- | --- |
| **Primary endpoints** |  |  |  |  |  |
| **Percent change**^b^ **in WHOQOL-OLD**^e^ **score** | 32.38  (27.60, 37.16) | 14.85  (10.86, 18.84) | 17.53  (11.48, 23.58) | -^f^ | <0.001^g^ |
| **Percent change**^b^ **in SAB**^h^ **score of the WHOQOL-OLD scale** | 85.77  (70.67, 100.87) | 36.18  (22.17, 50.19) | 49.59  (29.60, 69.59) | -^f^ | <0.001^g^ |
| **Percent change**^b^ **in AUT**^i^ **score of the WHOQOL-OLD scale** | 23.90  (14.25, 33.55) | 4.73  (-1.39, 10.85) | 19.17  (8.03, 30.30) | -^f^ | <0.001^g^ |
| **Percent change**^b^ **in DAD**^j^ **score of the WHOQOL-OLD scale** | 48.23  (29.06, 67.39) | 37.70  (6.26, 69.14) | 10.52  (-25.01, 46.05) | -^f^ | 0.148 |
| **Percent change**^b^ **in PPFA**^k^ **score of the WHOQOL-OLD scale** | 23.73  (14.50, 32.96) | 7.02  (2.07, 11.97) | 16.72  (6.50, 26.94) | -^f^ | <0.001^g^ |
| **Percent change**^b^ **in SP**^l^ **score of the WHOQOL-OLD scale** | 25.65  (16.46, 34.84) | 10.11  (4.43, 1578) | 15.54  (5.01, 26.07) | -^f^ | <0.001^g^ |
| **Percent change**^b^ **in INT**^m^ **score of the WHOQOL-OLD scale** | 34.13  (20.02, 48.23) | 39.13  (14.67, 63.60) | -5.01  (-32.25, 22.24) | -^f^ | 0.439 |
| **Secondary endpoints** |  |  |  |  |  |
| **Participants with ADL**^n^ **disability at 3-month follow-up, No. (%)** | 10 (37.04%) | 9 (47.37%) | -10.33  (-40.06, 19.40) | 0.53  (0.06, 3.47) | 0.514 |
| **Participants with IADL**^o^ **disability at 3-month follow-up, No. (%)** | 11 (40.74%) | 12 (63.16%) | -22.42  (-51.75, 6.9) | 0.34  (0.08, 1.32) | 0.130 |
| **Percent change**^b^ **in SSRS**^p^ **score** | 7.35  (2.57, 12.14) | -0.35  (-4.24, 3.54) | 7.70  (1.71,13.70) | -^f^ | 0.002^g^ |
| **Percent change**^b^ **in MFES**^q^ **score** | 8.61  (3.01, 14.21) | 8.10  (4.01, 12.19) | 5.12  (-6.23, 7.26) | -^f^ | 0.778 |
| **Percent change**^b^ **in ASHMAR**^r^ **score** | 11.89  (7.03, 16.76) | 4.22  (-2.69, 11.12) | 7.68  (-0.48, 15.84) | -^f^ | 0.002^g^ |

^a^SMART: Sensors and scales (receptor), a Mobile phone autonomous response system (central nervous system in the spinal cord), a Remote cloud management center (central nervous system in the brain), and a Total care system (effector).

^b^The percent change values are presented as “mean (95% CI)” values.

^c^Data are absolute differences between mean changes and expressed in percentage points.

^d^OR: odds ratio.

^e^WHOQOL-OLD: World Health Organization Quality of Life Instrument-Older Adults Module.

^f^Not applicable

^g^Indicate statistically significant variables (*P*<.05).

^h^SAB: sensory abilities.

^i^AUT: autonomy.

^j^DAD: death and dying.

^k^PPFA: past, present, and future activities.

^l^SP: social participation.

^m^INT: intimacy.

^n^ADL: activities of daily living.

^o^IADL: instrumental activities of daily living

^p^SSRS: Social Support Rating Scale.

^q^MFES: Modified Fall Efficacy Scale.

^r^AHSMSRS: The Rating Scale of Health Self-Management Skill for Adults.

**Supplementary results** **for the sensitivity analysis using relative change values as a measurement approach**

Table S11. Primary and secondary endpoints, along with the estimated differences observed in the intention-to-treat analysis when using relative change values as a measurement approach

| **Endpoints** | **SMART**^a^ **group (n=48)** | **Usual care group (n=46)** | **Difference**^c^ **(95% CI)^a^** | ***P value*** |
| --- | --- | --- | --- | --- |
| **Primary endpoints** |  |  |  |  |
| **Change**^b^ **in WHOQOL-OLD**^d^ **score** | 16.40  (14.70, 18.09) | 9.67  (8.17, 11.21) | 6.72  (4.47, 8.98) | <0.001^e^ |
| **Change**^b^ **in SAB**^f^ **score of the WHOQOL-OLD scale** | 5.63  (4.98, 6.27) | 2.91  (2.35, 3.48) | 2.71  (1.87, 3.56) | <0.001^e^ |
| **Change**^b^ **in AUT**^g^ **score of the WHOQOL-OLD scale** | 2.27  (1.67, 2.87) | 1.13  (0.60, 1.66) | 1.14  (0.35, 1.93) | <0.001^e^ |
| **Change**^b^ **in DAD**^h^ **score of the WHOQOL-OLD scale** | 3.08  (2.10, 4.06) | 2.41  (1.27, 3.56) | 0.67  (-0.82, 2.16) | 0.503 |
| **Change**^b^ **in PPFA**^i^ **score of the WHOQOL-OLD scale** | 2.38  (1.79, 2.96) | 1.17  (0.69, 1.66) | 1.20  (0.45,1.95) | <0.001^e^ |
| **Change**^b^ **in SP**^j^ **score of the WHOQOL-OLD scale** | 2.84  (2.16, 3.52) | 1.35  (0.89, 1.80) | 1.50  (0.69, 2.30) | <0.001^e^ |
| **Percent change**^b^ **in INT**^k^ **score of the WHOQOL-OLD scale** | 3.48  (2.57, 4.39) | 2.63  (1.72, 3.54) | 0.85  (-0.42, 2.11) | 0.395 |
| **Secondary endpoints** |  |  |  |  |
| **Change**^b^ **in SSRS**^l^ **score** | 2.13  (1.35, 2.90) | -0.09  (-0.89, 0.57) | 2.21  (1.11, 3.31) | <0.001^e^ |
| **Change**^b^ **in MFES**^m^ **score** | 0.59  (0.36, 0.82) | 0.40  (0.23, 0.57) | 0.19  (-0.09, 0.47) | 0.087 |
| **Change**^b^ **in ASHMAR**^n^ **score** | 6.13  (4.22, 8.04) | 3.01  (0.94, 5.08) | 3.12  (0.34, 5.90) | 0.002^e^ |

^a^SMART: Sensors and scales (receptor), a Mobile phone autonomous response system (central nervous system in the spinal cord), a Remote cloud management center (central nervous system in the brain), and a Total care system (effector).

^b^The percent change values are presented as “mean (95% CI)” values.

^c^Data are absolute differences between mean changes and expressed in percentage points.

^d^WHOQOL-OLD: World Health Organization Quality of Life Instrument-Older Adults Module.

^e^Indicate statistically significant variables (*P*<.05).

^f^SAB: sensory abilities.

^g^AUT: autonomy.

^h^DAD: death and dying.

^i^PPFA: past, present, and future activities.

^j^SP: social participation.

^k^INT: intimacy.

^l^SSRS: Social Support Rating Scale.

^m^MFES: Modified Fall Efficacy Scale.

^n^AHSMSRS: The Rating Scale of Health Self-Management Skill for Adults.

**Supplementary results** **of the per-protocol sensitivity analysis**

Table S12. Baseline characteristics of elderly participants in the per-protocol analysis (randomized controlled trial conducted from November 1 to December 31, 2020).

| **Variables** | **SMART**^a^ **Group**  **(n=41)** | **Usual Care Group**  **(n=42)** | **t/Z/χ2** | ***P value*** |
| --- | --- | --- | --- | --- |
| **Age** | 68.54 ± 5.07 | 69.81 ± 6.24 | 1.02^b^ | 0.311 |
| **Male** | 25 (60.98%) | 26 (61.90%) | 0.01^c^ | 0.931 |
| **BMI**^g^ **(kg/m^2^)** | 24.16 ± 4.01 | 23.29 ± 2.46 | -1.19^b^ | 0.239 |
| **Marital status** |  |  |  |  |
| **Married** | 26 (63.41%) | 34 (80.95%) | -^d, f^ | 0.108 |
| **Widowed** | 14 (34.15%) | 8 (19.05%) |  |  |
| **Divorced** | 1 (2.44%) | 0 (0.00%) |  |  |
| **Education** |  |  | 2.09^e^ | 0.555 |
| **Primary school and below** | 9 (21.95%) | 5 (11.90%) |  |  |
| **Junior high school** | 10 (24.39%) | 13 (30.95%) |  |  |
| **Senior high or vocational school** | 15 (36.59%) | 14 (33.33%) |  |  |
| **College and above** | 7 (17.07%) | 10 (23.81%) |  |  |
| **Dwelling status** |  |  | -^d, f^ | 0.366 |
| **Living with others** | 39 (95.12%) | 41 (97.62%) |  |  |
| **Living alone** | 2 (4.88%) | 0 (0.00%) |  |  |
| **Nursing home** | 0 (0.00%) | 1 (2.38%) |  |  |
| **Visual impairment** | 17 (41.46%) | 17 (40.48%) | 0.01^c^ | 0.927 |
| **Hearing impairment** | 17 (41.46%) | 16 (38.10%) | 0.10^c^ | 0.754 |
| **Smoking** | 12 (29.27%) | 8 (19.05%) | 1.19^c^ | 0.276 |
| **Alcohol drinking** | 9 (21.95%) | 5 (11.90%) | 1.49^c^ | 0.222 |

^a^SMART: Sensors and scales (receptor), a Mobile phone autonomous response system (central nervous system in the spinal cord), a Remote cloud management center (central nervous system in the brain), and a Total care system (effector).

^b^Student t test.

^c^chi-square test.

^d^Fisher exact test.

^e^Wilcoxon rank-sum test.

^f^not applicable.

^g^BMI, body mass index.

Figure S7. Effects of the personalized and integrated care provided by the SMART system combined with usual care versus usual care on the primary and secondary endpoints in the per-protocol analysis. (A) Effects of the personalized and integrated care provided by the SMART system combined with usual care versus usual care on quality of life in the per-protocol analysis. (B) Effects of the personalized and integrated care provided by the SMART system combined with usual care versus usual care on functional status in the per-protocol analysis. (C) Effects of the personalized and integrated care provided by the SMART system combined with usual care versus usual care on other secondary outcomes in the per-protocol analysis. WHOQOL-OLD, World Health Organization Quality of Life Instrument-Older Adults Module; SAB, Sensory Abilities; AUT, Autonomy; DAD, Death and Dying; PPFA, Past, Present, and Future Activities; SP, Social Participation; INT, Intimacy; ADL, Activities of Daily Living; IADL, Instrumental Activities of Daily Living; SSRS, Social Support Rating Scale; MFES, Modified Fall Efficacy Scale; AHSMSRS, The Rating Scale of Health Self-Management Skill for Adults.

Table S13. Primary and secondary endpoints, along with the estimated differences observed in the per-protocol analysis

| **Endpoints** | **SMART**^a^ **group (n=41)** | **Usual care group (n=42)** | **Difference**^c^ **(95% CI)^a^** | **OR**^d^ **(95%CI)** | ***P value*** |
| --- | --- | --- | --- | --- | --- |
| **Primary endpoints** |  |  |  |  |  |
| **Percent change**^b^ **in WHOQOL-OLD**^e^ **score** | 27.82  (24.15, 31.59) | 16.89  (14.05, 19.73) | 10.98  (6.37, 15.59) | -^f^ | <0.001^g^ |
| **Percent change**^b^ **in SAB**^h^ **score of the WHOQOL-OLD scale** | 81.06  (69.58, 92.54) | 40.86  (31.24, 50.48) | 40.20  (25.45, 54.94) | -^f^ | <0.001^g^ |
| **Percent change**^b^ **in AUT**^i^ **score of the WHOQOL-OLD scale** | 17.26  (12.71, 21.81) | 9.63  (5.58, 13.68) | 7.63  (1.63, 13.63) | -^f^ | <0.001^g^ |
| **Percent change**^b^ **in DAD**^j^ **score of the WHOQOL-OLD scale** | 36.18  (22.48, 49.88) | 36.20  (17.89, 54.51) | -0.02  (-22.55, 22.51) | -^f^ | 0.997 |
| **Percent change**^b^ **in PPFA**^k^ **score of the WHOQOL-OLD scale** | 19.11  (12.77, 25.44) | 10.24  (6.55, 13.94) | 8.86  (1.64, 16.09) | -^f^ | <0.001^g^ |
| **Percent change**^b^ **in SP**^l^ **score of the WHOQOL-OLD scale** | 23.85  (16.58, 31.13) | 12.80  (8.66, 16.96) | 11.05  (2.80, 19.29) | -^f^ | <0.001^g^ |
| **Percent change**^b^ **in INT**^m^ **score of the WHOQOL-OLD scale** | 32.69  (21.73, 43.66) | 31.17  (17.91, 43.66) | 1.52  (-15.43, 18.47) | -^f^ | 0.746 |
| **Secondary endpoints** |  |  |  |  |  |
| **Participants with ADL**^n^ **disability at 3-month follow-up, No. (%)** | 18 (43.90%) | 23 (54.76%) | -8.65  (-30.04, 12.74) | 0.57  (0.12, 2.53) | 0.463 |
| **Participants with IADL**^o^ **disability at 3-month follow-up, No. (%)** | 14 (34.15%) | 20 (47.62%) | -15.68  (-37.25, -5.89) | 0.34  (0.12, 0.92) | 0.038^g^ |
| **Percent change**^b^ **in SSRS**^p^ **score** | 6.57  (3.18, 9.96) | 1.00  (0.27, 1.72) | 5.57  (2.16, 8.98) | -^f^ | <0.001^g^ |
| **Percent change**^b^ **in MFES**^q^ **score** | 8.07  (4.15, 11.99) | 5.74  (3.08, 8.41) | 2.33  (-2.34, 7.00) | -^f^ | 0.291 |
| **Percent change**^b^ **in ASHMAR**^r^ **score** | 10.87  (7.08, 14.66) | 3.91  (0.63, 7.19) | 6.96  (2.03, 11.90) | -^f^ | <0.001^g^ |

^a^SMART: Sensors and scales (receptor), a Mobile phone autonomous response system (central nervous system in the spinal cord), a Remote cloud management center (central nervous system in the brain), and a Total care system (effector).

^b^The percent change values are presented as “mean (95% CI)” values.

^c^Data are absolute differences between mean changes and expressed in percentage points.

^d^OR: odds ratio.

^e^WHOQOL-OLD: World Health Organization Quality of Life Instrument-Older Adults Module.

^f^Not applicable

^g^Indicate statistically significant variables (*P*<.05).

^h^SAB: sensory abilities.

^i^AUT: autonomy.

^j^DAD: death and dying.

^k^PPFA: past, present, and future activities.

^l^SP: social participation.

^m^INT: intimacy.

^n^ADL: activities of daily living.

^o^IADL: instrumental activities of daily living

^p^SSRS: Social Support Rating Scale.

^q^MFES: Modified Fall Efficacy Scale.

^r^AHSMSRS: The Rating Scale of Health Self-Management Skill for Adults.

**References**

1. Austin, P. C., Lee, D. S., Ko, D. T., & White, I. R. (2019). Effect of Variable Selection Strategy on the Performance of Prognostic Models When Using Multiple Imputation. Circulation. Cardiovascular quality and outcomes, 12(11), e005927. https://doi.org/10.1161/CIRCOUTCOMES.119.005927
2. Beesley, L. J., & Taylor, J. M. G. (2021). A stacked approach for chained equations multiple imputation incorporating the substantive model. Biometrics, 77(4), 1342-1354. https://doi.org/10.1111/biom.13372
3. White, I. R., Royston, P., & Wood, A. M. (2011). Multiple imputation using chained equations: Issues and guidance for practice. Statistics in medicine, 30(4), 377-399. https://doi.org/10.1002/sim.4067
